# Balancing Oncological Care and Quality of Life: Diagnostic and Therapeutic Strategies for Chemotherapy-Induced Cardiotoxicity—A Narrative Review

**DOI:** 10.3390/jcm15176760

**Published:** 2026-08-31

**Authors:** Alexandra Huiduc, Ovidiu Popa-Velea, Adriana Mihaela Ilieșiu

**Affiliations:** 1 Department of Medical Semiology, Faculty of Medicine, “Carol Davila” University of Medicine and Pharmacy, 020021 Bucharest, Romania; alexandra.huiduc@drd.umfcd.ro; 2 Department of Medical Psychology, Faculty of Medicine, “Carol Davila” University of Medicine and Pharmacy, 020021 Bucharest, Romania; 3 Department of Cardiology, “Prof. Dr. Theodor Burghele” Clinical Hospital, Carol Davila University of Medicine and Pharmacy, 020021 Bucharest, Romania; adriana.iliesiu@umfcd.ro

**Keywords:** cancer, cardiotoxicity, chemotherapy, cardio-oncology, quality of life

## Abstract

**Background**: Cardiovascular complications of cancer therapy may affect patients far beyond conventional measures of cardiac function, yet their relationship with health-related quality of life (HRQoL) remains incompletely defined. This review examined how cardiovascular status and cardiotoxicity-related interventions relate to HRQoL across cancer treatment and survivorship. **Methods**: Twenty-one studies were reviewed and reassessed according to four research questions: established cardiovascular disease or cardiac dysfunction and HRQoL; subclinical cardiovascular abnormalities and subsequent HRQoL; interventions targeting cardiotoxicity with HRQoL assessment; and supportive interventions improving HRQoL without demonstrated cardioprotection. Evidence was additionally considered across diagnostic, pharmacological/cardioprotective, exercise, and supportive-care domains. **Results**: The available evidence revealed a clinically relevant pattern. Established cardiovascular disease or cardiac dysfunction showed the clearest association with poorer HRQoL, particularly in physical domains, whereas evidence linking subclinical abnormalities to subsequent patient-reported deterioration was sparse. Most studies evaluated interventions, including cardioprotective pharmacological strategies and exercise-based approaches, but improvements in HRQoL were not consistently accompanied by, or attributable to, demonstrated cardioprotection. Exercise and supportive interventions were also associated with improvements in functional or patient-reported outcomes in some studies, independently of demonstrable cardiovascular benefit. Across domains, substantial heterogeneity in populations, cardiovascular measures, interventions, HRQoL instruments, and study designs limited direct comparison and causal interpretation. **Conclusions**: Cardiovascular health and HRQoL appear closely interconnected, but the available evidence does not establish a consistent link between cardiovascular injury and subsequent changes in HRQoL. The literature is richer in prevention and intervention studies than in longitudinal assessment of the patient-reported consequences of cardiovascular injury. Larger prospective studies incorporating standardized serial cardiovascular assessment and HRQoL as a predefined primary endpoint are needed to establish temporal relationships and clinically meaningful cardiovascular determinants of HRQoL.

## 1. Introduction

Over the past decades, oncological care has undergone profound transformations, resulting in significant improvements in patient survival, with large-scale epidemiological data indicating 5-year survival rates exceeding 65–70% across many cancer types [1,2]. These improvements are not solely attributable to earlier diagnosis, but also to major therapeutic advances, including the refinement of conventional treatments and the introduction of novel modalities. In particular, immunotherapy—most notably immune checkpoint inhibitors—and adoptive cell transfer (ACT) approaches, including CAR-T cell therapy, have significantly advanced the treatment of various malignancies such as melanoma, lung cancer, and hematologic cancers, resulting in strong therapeutic responses and, in some cases, durable long-term remission [3,4].

Despite these therapeutic breakthroughs, chemotherapy remains the backbone of systemic anticancer treatment, reflecting its established efficacy, broad indications, and continued indispensability in clinical practice, particularly where novel therapies are unavailable or not indicated. Population-based analyses suggest that approximately 50–60% of cancer patients receive chemotherapy at some point during their disease course, whereas immunotherapy remains currently applicable only to a significantly smaller proportion of patients, often estimated at below 20–30%, depending on tumour type and biomarker eligibility [5,6].

The widespread use of chemotherapy across diverse tumour types and disease stages makes the management of its associated toxicities essential, as chemotherapy-induced adverse effects—including fatigue, nausea, neurological impairment, and cardiotoxicity—can significantly impair daily functioning, well-being and overall health-related quality of life (HRQoL) [7]. Among these, cardiotoxicity represents one of the most serious complications, particularly in the case of anthracyclines. The incidence of anthracycline-induced cardiotoxicity has been reported to range between 9% and 26%, depending on cumulative dose and patient-related risk factors [8]. Importantly, these cardiovascular complications may manifest years after treatment completion, contributing to long-term morbidity in cancer survivors.

Recognition that anthracycline- and other cancer therapy–related cardiovascular toxicities could undermine survival gains achieved with modern anticancer treatments prompted multidisciplinary strategies to preserve cardiovascular and oncological outcomes. This need drove the emergence of cardio-oncology, promoting prevention, early detection, and patient-centred care through collaboration among cardiologists, oncologists, haematologists, imaging specialists, and other healthcare professionals [9,10,11]. The discipline has been supported by scientific societies, including the International Cardio-Oncology Society (ICOS), and major meetings organized by the European Society of Cardiology (ESC), American College of Cardiology (ACC), and ICOS, fostering research collaboration and evidence-based practice [9,10,11]. These efforts culminated in the 2022 ESC Guidelines on Cardio-Oncology, establishing a comprehensive framework for cardiovascular risk stratification, surveillance, prevention, and management, while introducing refined definitions such as “cancer therapy–related cardiac dysfunction” (CTRCD) and recommending biomarkers and advanced imaging for early detection [11,12,13]. In particular, cardiac troponins and N-terminal pro-B-type natriuretic peptide (NT-proBNP) have been validated as early indicators of myocardial injury [14]. In parallel, techniques such as global longitudinal strain (GLS), assessed by speckle-tracking echocardiography, have demonstrated superior sensitivity compared to conventional left ventricular ejection fraction (LVEF) in detecting subclinical myocardial dysfunction [15]. Preventive strategies have also shown promise; for example, the use of cardioprotective agents, such as beta-blockers, angiotensin-converting enzyme inhibitors, and dexrazoxane, has been associated with a reduction in the incidence and severity of CTRCD [16,17].

Despite these advances, an important gap remains in both the literature and current clinical guidelines. Although cardio-oncology has substantially improved the understanding of the pathophysiology, diagnosis, prevention, and management of cancer therapy–related cardiotoxicity, the effects of these strategies on patients’ HRQoL remain poorly understood, largely because many studies to date have focused on traditional clinical endpoints (such as changes in cardiac function or the incidence of cardiovascular events), rather than evaluating how these outcomes affect patients’ daily functioning and overall well-being. In this context, the 2022 ESC Guidelines acknowledge the limited availability of robust evidence regarding patient-reported outcomes (PROs) and HRQoL [11].

Addressing this gap is increasingly important because HRQoL has become a fundamental outcome in contemporary cancer care. Both cancer and cardiovascular disease independently impair physical, emotional, and social functioning [18,19], and their coexistence in cardio-oncology patients is likely to result in an even greater cumulative burden. Consequently, the evaluation of diagnostic, preventive, and therapeutic cardio-oncology strategies should extend beyond cardiovascular outcomes to include their effects on patient-reported outcomes, functional status, and HRQoL.

In this context, the present study seeks to investigate chemotherapy-related cardiotoxicity from an HRQoL perspective, focusing on the role of current diagnostic modalities in the early detection of cardiotoxicity among cancer patients undergoing treatment, as well as the impact of cardioprotective strategies on patient-reported HRQoL. Such an approach is essential not only for optimizing individualized patient care by improving well-being and treatment satisfaction, but also for informing healthcare policy and resource allocation. Improved HRQoL has been associated with reduced healthcare utilization and costs, while also contributing to sustained societal productivity.

## 2. Materials and Methods

This study was designed as a narrative review to provide an integrated overview of the current evidence on chemotherapy-induced cardiotoxicity and its relationship with HRQoL. Given the substantial heterogeneity of the available literature—including differences in study designs, HRQoL assessment instruments, diagnostic modalities for cardiotoxicity, chemotherapy regimens, and cardioprotective interventions—a narrative synthesis was considered the most appropriate methodological approach. To improve transparency and ensure a structured process for identifying relevant evidence, literature searches were conducted in PubMed, Web of Science, and Scopus using predefined search terms and eligibility criteria. The search strategy was structured around four core concepts—”health-related quality of life” (HRQoL), “cardiotoxicity”, “cancer therapy-related cardiac dysfunction” (CTRCD), and “cancer/neoplasm”. Within each concept domain, synonyms and related terms were combined using the Boolean operator OR, while the core concept domains were combined using AND. The search strategy was adapted to the syntax and search fields of each database. Full database-specific search strings and search dates are provided in Section S1. The literature search was initiated in June 2025 and subsequently updated, with the final database search performed in January 2026.

No restrictions were applied regarding publication date or article type during the initial search. Following duplicate removal, titles and abstracts were screened according to the predefined eligibility criteria to identify English-language original studies conducted in adult participants (≥18 years) receiving at least chemotherapy, either alone or in combination with other systemic anticancer therapies. Gray literature, studies evaluating treatment modalities other than chemotherapy, investigations focusing on adverse effects unrelated to chemotherapy-induced cardiotoxicity, and studies that did not examine the diagnosis, prevention, or clinical impact of chemotherapy-induced cardiotoxicity in relation to HRQoL were excluded. When review articles or meta-analyses were identified, their reference lists were manually screened to identify additional relevant original studies.

Study selection was performed independently by two investigators (A.H. and O.P.-V.). Any discrepancies were resolved through discussion and, when necessary, by consultation with a third investigator (A.M.I.).

Studies were considered eligible if they addressed at least one of the predefined research questions (RQ) concerning the relationship between cardiovascular status, cardiotoxicity, interventions, and HRQoL. These research questions were defined as follows:

RQ 1: What is the association between documented cardiac injury or cardiovascular disease and HRQoL? RQ 2: What is the association between imaging or biomarker abnormalities and subsequent HRQoL? RQ 3: Which interventions prevent or treat cardiotoxicity while also assessing HRQoL? RQ 4: Which general supportive interventions improve HRQoL without demonstrated cardioprotection?

Each included study was subsequently assigned to the research question that best reflected its primary focus and contribution to the available evidence. This classification distinguished studies directly addressing the relationship between cardiovascular injury or dysfunction and HRQoL from those evaluating interventions that may improve HRQoL independently of demonstrated cardioprotection.

**Figure 1:**
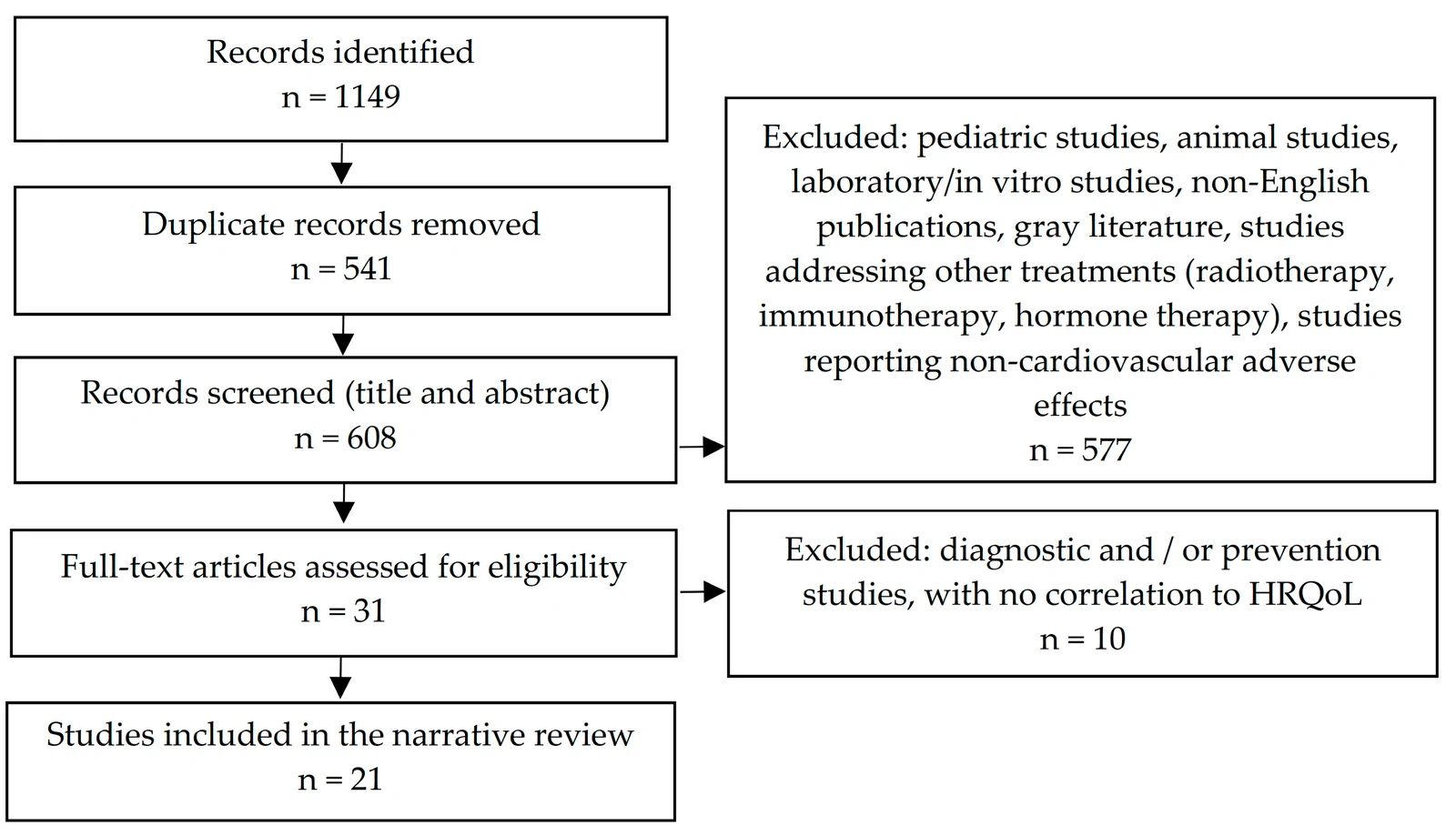
Flow diagram of the literature search and study selection process.

Following the selection process, 21 articles were included in the review from the 1149 records initially identified. A summary of the complete selection procedure is depicted in Figure 1.

## 3. Results

### 3.1. Study Selection and Characteristics

Table 1 provides an overview of studies investigating diagnostic strategies for chemotherapy-related cardiotoxicity and their association with quality of life (QoL).

All studies evaluated HRQoL and focused on interventions for the diagnosis or prevention of chemotherapy-induced cardiotoxicity, with several also incorporating psychological assessment tools. Although the majority of papers were from the past five years, highlighting the growing interest in this area, the first article on HRQoL related to chemotherapy-induced cardiotoxicity dates back to 2004 [26]. The studies spanned multiple continents and included diverse demographic populations. Mostly, the papers were centred on breast cancer, reflecting its global prevalence and the well-known cardiotoxic risk of its treatments [43,44]. Eighteen of the 21 studies focused on patients who had received anthracycline-containing chemotherapy regimens. The sample sizes in the studies ranged from 2 to 272 participants. Smaller sample sizes were often observed in studies adopting interdisciplinary oncological, cardiological, and psychological perspectives.

The wide variety of HRQoL instruments used across studies reflects the methodological and conceptual diversity in how well-being is measured. This diversity offers a broad perspective on different HRQoL domains addressed by each tool, contributing to a comprehensive understanding of the needs of oncology patients.

Additionally, we identified substantial variability in study designs, ranging from case studies and qualitative research to more traditional quantitative studies. Given this heterogeneity, a narrative synthesis was deemed more appropriate than alternative approaches, as it allows for a flexible and comprehensive integration of findings and is particularly well suited to mapping conceptual frameworks in emerging fields such as this one.

#### Instruments Assessment

HRQoL was evaluated using a combination of generic and disease-specific instruments, enabling both broad assessment of overall health status and targeted evaluation of disease-related effects. Detailed information is provided in Section S2 (Tables S1–S3).

The most commonly used tool for generic HRQoL assessment was the Short Form 36 Health Survey (SF-36), used in seven studies [20,22,23,28,31,33,41]. Two of these studies [28,41] referred to it as the “Medical Outcomes Study SF-36”.

SF-36 is a multidimensional instrument [45] including eight domains: Physical Functioning (PF), Role Physical (RP), Bodily Pain (BP), General Health (GH), Mental Health (MH), Role Emotional (RE), Vitality (VT), and Social Functioning (SF). The first four domains reflect physical health, the next three measure mental health, and the final domain focuses on social functioning.

The main advantage of the SF-36 is that it captures HRQoL from multiple dimensions, unlike single-index instruments, allowing for a more comprehensive assessment of health status (e.g., EQ-5D-5L). It allows for detailed detection of specific deficits in different domains (e.g., physical pain vs. emotional well-being), which is critical for populations with complex or chronic conditions. Moreover, it tracks subtle changes in both physical and mental health. While other tools may only identify overall HRQoL impairment, the SF-36 pinpoints which domains are specifically affected, offering more precise monitoring of interventions. Its strong psychometric properties—internal consistency and construct validity across languages, cultures, and clinical contexts—provide a robust empirical foundation for clinical applications.

The SF-36 yields two main scores: the Physical Component Summary (PCS) and the Mental Component Summary (MCS). Both scores are positively correlated with overall HRQoL. A higher PCS score indicates better physical functioning, less pain, fewer role limitations due to physical problems, and better general health. Similarly, a higher MCS score reflects better mental well-being, greater vitality, fewer emotional limitations, and better social functioning.

Several studies used disease-specific instruments to complement generic HRQoL measures, providing greater clinical sensitivity to condition-specific symptoms, treatment effects, and clinically relevant dimensions not fully captured by global HRQoL scales. These were particularly relevant in oncology and cardiology:

- in oncological populations, commonly used instruments included the Functional Assessment of Cancer Therapy–General, the European Organisation for Research and Treatment of Cancer Quality of Life Questionnaire–Core 30, and related modules such as Functional Assessment of Cancer Therapy–Lymphoma and Functional Assessment of Cancer Therapy–Breast. Symptom-focused tools such as Functional Assessment of Chronic Illness Therapy–Fatigue and EORTC Quality of Life Questionnaire–Fatigue 12-item module were also frequently applied, particularly for cancer-related fatigue;
- in cardiac populations, HRQoL was primarily assessed using the Minnesota Living with Heart Failure Questionnaire and the M.D. Anderson Symptom Inventory–Heart Failure module, both of which capture symptom burden and functional limitations specific to heart failure.

Several studies also included supporting clinical and functional tools—such as the Karnofsky and Eastern Cooperative Oncology Group performance status scales—as well as symptom- or behaviour-related measures (e.g., fatigue, physical activity, psychological distress). While not HRQoL instruments *per se*, they provide important contextual information for interpreting PROs.

Overall, the heterogeneity of instruments reflects the multidimensional nature of HRQoL but limits direct comparability across studies, requiring results to be interpreted within the context of each instrument.

### 3.2. Research Questions Addressed by the Included Studies

Reassessment of the 21 included studies according to the four research questions revealed substantial heterogeneity in the nature of the cardiovascular–HRQoL evidence. The studies did not address a single pathway linking cardiotoxicity to HRQoL, but rather examined different stages of cardiovascular involvement, ranging from established cardiovascular disease or cardiac dysfunction to subclinical abnormalities detected through objective cardiovascular assessment, as well as interventions intended to prevent or mitigate cardiotoxicity and general supportive interventions. This distinction is particularly relevant when interpreting exercise-based studies, in which improvements in HRQoL may occur independently of a demonstrable cardioprotective effect.

RQ1: Association between documented cardiac injury or cardiovascular disease and HRQoL

Two studies addressed the relationship between established cardiovascular involvement and HRQoL. Lubberts et al. [23] evaluated long-term cardiovascular disease in testicular cancer survivors and found that survivors with cardiovascular disease reported poorer QoL, particularly in physical domains, than those without cardiovascular disease. Koop et al. [42] similarly examined HRQoL in breast cancer patients with cardiac dysfunction, directly relating PROs to established cardiac impairment. Together, these studies provide the most direct evidence within the included literature that clinically manifest cardiovascular disease or cardiac dysfunction is associated with impaired HRQoL. Importantly, these findings demonstrate an association rather than establishing that cardiovascular disease is the sole or direct cause of impaired HRQoL.

RQ2: Association between imaging or biomarker abnormalities and subsequent HRQoL

Three studies provided data relevant to objectively detected cardiovascular abnormalities and their potential implications for HRQoL, making this the least extensively represented cardiovascular evidence domain, despite its potential relevance for the early detection of cardiotoxicity. Among these, Geršak et al. [22] directly evaluated the relationship with HRQoL in a comprehensive longitudinal assessment incorporating clinical cardiotoxicity, cardiac biomarkers, echocardiographic parameters, myocardial strain assessed by fast strain-encoded (fast-SENC) cardiac magnetic resonance (CMR), and QoL measures. The other two studies provide more contextual evidence: Drafts et al. [21] demonstrated that low-to-moderate cumulative anthracycline exposure was associated with early, non-invasive imaging evidence of subclinical cardiovascular disease and also incorporated QoL assessment, highlighting the presence of measurable cardiovascular abnormalities before the onset of overt clinical disease; Peck et al. [37] used QoL measures alongside assessments of physical activity, cardiac function, and cardiorespiratory fitness in women with human epidermal growth factor receptor 2 (HER2)-positive breast cancer. We considered these two studies as providing contextual evidence rather than direct evidence of an association between objectively detected cardiovascular abnormalities and subsequent HRQoL.

Collectively, these studies suggest that cardiovascular abnormalities detectable before or alongside clinically apparent disease may be relevant to PROs. However, the small number of studies addressing this question indicates that the temporal relationship between subclinical cardiovascular abnormalities and subsequent HRQoL remains insufficiently characterized.

RQ3: Interventions that prevent or treat cardiotoxicity and also assess HRQoL

This represented the largest evidence domain, encompassing all six studies in the pharmacological and cardioprotective section and six of the ten exercise-based studies. The pharmacological studies examined different strategies for modifying treatment-related cardiovascular risk, while incorporating HRQoL or PROs. Huang et al. [24] evaluated liposomal doxorubicin within an ifosfamide-based regimen and reported favourable effects on cardiotoxicity together with HRQoL outcomes, whereas Sideras et al. [25] evaluated QoL in the context of an anthracycline-sparing pixantrone strategy, without demonstrating a significant difference in QoL change over time. Ganz et al. [28] examined long-term cardiac function and QoL following trastuzumab-containing therapy, while Suntheralingam et al. [27] addressed the clinically relevant question of continuing trastuzumab in patients with mild cardiotoxicity. Conte et al. [26] and Skrypnyk et al. [31] likewise assessed treatment-related cardiovascular considerations together with PROs. Within the exercise literature, Heinze-Milne et al. [32], Hughes et al. [33], Costa et al. [34], Murray et al. [35], Schneider et al. [36], and Díaz-Balboa et al. [38] evaluated exercise or cardio-oncology rehabilitation in populations receiving or exposed to potentially cardiotoxic treatment, with cardiovascular or functional outcomes considered alongside HRQoL or related PROs. Nevertheless, the included studies varied substantially in whether cardioprotection was directly demonstrated. Accordingly, an improvement in HRQoL following an intervention should not, by itself, be interpreted as evidence that cardioprotection mediated the observed benefit.

RQ4: General supportive interventions that improve HRQoL without demonstrated cardioprotection

Four studies were classified under RQ4. These studies primarily evaluated exercise or supportive interventions in relation to physical function, symptoms, fitness, or HRQoL, without demonstrating that prevention or attenuation of cardiac injury accounted for the observed PROs. Buziashvili et al. [20] assessed QoL during neoadjuvant chemotherapy, while Vincent et al. [39], Spector et al. [40], and Griffith et al. [41] evaluated exercise-based approaches in cancer populations. These studies illustrate the distinction emphasized by the present analytical framework: exercise and other supportive interventions may improve physical functioning, fitness, symptoms, or QoL through direct non-cardiovascular mechanisms. Such findings therefore cannot be used to infer cardioprotection in the absence of objective evidence demonstrating a cardiovascular effect.

Taken together, the four research questions reveal a clinically relevant gradient in the available evidence. Studies involving established cardiovascular disease or cardiac dysfunction (RQ1) provide relatively direct evidence of an association with HRQoL, whereas studies addressing objectively detectable, potentially subclinical cardiovascular abnormalities (RQ2) are fewer and more heterogeneous. The intervention literature is considerably more extensive, but it requires a clear distinction between interventions explicitly targeting cardiotoxicity (RQ3) and supportive interventions that improve HRQoL without demonstrating cardioprotection (RQ4). Thus, the available evidence indicates an association between cardiovascular status and HRQoL throughout different stages of cancer treatment and survivorship; however, it does not support the assumption that improvements in HRQoL following exercise, pharmacological treatment, or other interventions are consistently mediated through the prevention or reduction of cardiac injury.

**Section A. Diagnostic studies**

Drafts et al. [21] provided evidence that deterioration in LVEF and increased left ventricular end-systolic volume, along with impaired myocardial contractility (decreased mid-wall circumferential strain), as detected by CMR imaging, occurred alongside worsening HRQoL. These cardiac and vascular changes occurred independently of factors such as age, gender, race, cumulative anthracycline dose, administration technique, cancer type, and pre-existing cardiovascular conditions. Furthermore, structural and functional changes observed on CMR were accompanied by a deterioration in HRQoL, as reflected by an increase in the Minnesota Living with Heart Failure Questionnaire score from approximately 15.4 to 28.5. Biomarkers such as troponin I and B-type natriuretic peptide did not show significant overall elevations, although some subclinical increases in troponin I were noted. Importantly, changes in QoL were not significantly associated with changes in LVEF or myocardial strain, suggesting a temporal association rather than a direct relationship between cardiac abnormalities and HRQoL.

Symptoms such as weakness, dyspnoea, and reduced exercise tolerance [20], as well as the indication for cardioprotective therapy [22], were significantly associated with poorer QoL, with the latter being associated with poorer physical and mental HRQoL. Lubberts et al. (2023) [23] reported that cancer survivors developed cardiovascular disease during long-term follow-up. The study identified several risk factors for cardiovascular disease, including platinum-based chemotherapy (compared to orchidectomy alone), obesity at the time of testicular cancer diagnosis, smoking at diagnosis, Raynaud’s phenomenon, dyslipidaemia, and a family history of cardiovascular disease. Survivors who developed cardiovascular disease reported reduced HRQoL in several physical domains, manifesting as lower vitality, increased fatigue, and more bodily pain. However, there was no significant difference in mental health or social functioning between survivors with and without cardiovascular disease.

**Section B. Cardioprotective pharmacological studies**

To preserve HRQoL and protect cardiovascular health in cancer patients, several strategies have been explored regarding the selection and administration of chemotherapeutic regimens. These strategies include modifications in the molecular structure of chemotherapeutic agents, alterations in treatment schedules, the choice between concomitant and sequential administration, and the use of cardioprotective strategies. Among these approaches, the use of liposomal doxorubicin instead of conventional doxorubicin, in combination with ifosfamide, was associated not only with a lower incidence of cardiotoxicity, but also with superior antitumor efficacy. This regimen also showed higher HRQoL scores across several QLQ-C30 domains, including physical, role, emotional, cognitive, and social functioning [24]. For epirubicin and paclitaxel, administered either concomitantly or sequentially [26], no clear differences in QoL were identified between treatment strategies. Although cardiac events, including grade II cardiotoxicity, occurred in the concomitant arm, the QoL analyses did not demonstrate a clear advantage for either regimen. Two studies examined the integration of trastuzumab into chemotherapeutic regimens. Suntheralingam et al. (2024) [27] showed that when trastuzumab was administered sequentially after anthracycline therapy, treatment could continue safely in patients with mild cardiotoxicity under cardio-oncology management with angiotensin-converting enzyme inhibitors and/or beta-blockers. No significant differences in HRQoL or CMR-derived measures, including LVEF, ventricular volumes, and markers of myocardial oedema or fibrosis, were observed between patients in whom trastuzumab was continued and those in whom treatment was interrupted. Ganz et al. [28] compared adjuvant anthracycline/taxane chemotherapy with and without trastuzumab. In patients without pre-existing cardiovascular comorbidities, no significant differences in cardiac function or HRQoL were found. However, older age and prior antihypertensive medication use were associated with lower functional capacity, as assessed by the Duke Activity Status Index.

Finally, Skrypnyk et al. [31] examined L-arginine as a strategy for preventing anthracycline-associated myocardial injury in patients with acute leukaemia and concomitant ischemic heart disease. L-arginine administration was associated with a lower risk of anthracycline-induced myocardial injury and improved SF-36 outcomes, particularly in patients with concomitant ischemic heart disease. However, the study did not directly demonstrate that the improvement in HRQoL resulted from prevention of cardiac injury.

**Section C. Exercise studies**

Ten studies investigated physical activity as an intervention strategy; however, the exercise protocols and reported outcomes were highly heterogeneous. The characteristics of the physical activity interventions varied substantially across the included studies, particularly with regard to exercise modality, intensity, frequency, and duration. Table 2 summarizes the physical activity regimens evaluated for the prevention of chemotherapy-induced cardiotoxicity, together with relevant details regarding the structure and implementation of each intervention.

One study with a small sample size (n = 2) [33] found preliminary evidence of beneficial effects of physical activity after a 16-week individualized exercise program, consisting of progressive training performed three times a week for about 30 min per session, several years after chemotherapy completion. Improvements in cardiac performance were seen in one participant, while the other participant maintained cardiac function. Specifically, cardiorespiratory fitness (VO_2_ peak) increased from 13.9 to 14.3 mL O_2_/kg/min in Participant 1 and from 12.5 to 18.7 mL O_2_/kg/min in Participant 2. LVEF improved from about 25–30% to 35–40% in Participant 2, while it remained stable in Participant 1. Both participants reported improvements in mental HRQoL, and one showed meaningful gains in physical HRQoL, as measured by the SF-36 physical and mental component scales. Given the two-participant case-series design, these findings should be interpreted as preliminary rather than as evidence of efficacy.

Peck et al. (2022) [37] examined self-reported physical activity, rather than structured exercise programs, with interesting results. Specifically, early declines in moderate-to-vigorous physical activity (MVPA), HRQoL, and cardiac function were observed following anthracycline treatment and during the initial months of trastuzumab therapy. Although MVPA was not associated with recovery of cardiac function, which remained below baseline at the end of treatment, higher levels of MVPA during treatment were associated with more favourable cardiac function parameters, including global longitudinal strain (GLS) and the E/A ratio.

In contrast, the study by Heinze Milne et al. (2021) [32] found that a 12-week supervised aerobic exercise program during anthracycline therapy was feasible, but did not result in significant changes in HRQoL or cardiotoxicity-related biomarkers. Thus, the feasibility of exercise during treatment did not translate into demonstrable improvements in either PROs or the measured cardiovascular biomarkers.

Regarding the timing of exercise initiation in relation to chemotherapy, Schneider et al. (2024) [36] observed that patients who engaged in exercise training during anthracycline-based chemotherapy had a moderate decline in cardiorespiratory fitness (VO_2_ peak), compared to baseline values. Conversely, patients who started exercise after chemotherapy completion showed improvements in VO_2_ peak, with median increases of approximately 2.8 mL/kg/min. Interestingly, patients on non-anthracycline regimens (either cardiotoxic or non-cardiotoxic chemotherapy) demonstrated improvements in VO_2_ peak when exercise was performed during treatment. However, neither the timing of exercise nor the chemotherapy regimen had a clear impact on HRQoL outcomes, although higher baseline HRQoL scores were associated with smaller changes over time.

Vincent et al. (2020) [39] suggested that clinicians should consider recommending physical activity during chemotherapy, rather than waiting until after treatment. Initiating exercise during therapy appeared to reduce the typical decline in cardiorespiratory fitness during treatment, providing greater short-term benefits compared to starting after chemotherapy. Early physical activity also helped counteract treatment-related deconditioning, including fatigue, reduced aerobic capacity, and physical weakness. However, these differences diminished by the 12-month follow-up. Overall, HRQoL remained stable throughout the study, with no significant differences between groups, based on the timing of exercise. Emotional functioning showed a transient benefit, and although anxiety levels either increased or remained stable, depression scores tended to improve.

The exercise programs varied widely, including aerobic exercise alone or combined with resistance training, delivered either in supervised clinical settings or through home-based regimens. Aerobic exercise was the main intervention in four studies and a component of multicomponent programs in five others. This heterogeneity is relevant when interpreting HRQoL outcomes, as exercise may influence PROs through direct functional, symptomatic, and psychosocial pathways that are not necessarily mediated by cardiovascular effects.

Murray et al. (2023) [35] reported mixed responses to aerobic exercise during dose-dense anthracycline chemotherapy. Some participants showed reductions in VO_2_ peak, while others maintained or improved it by the end of treatment. Moderate declines in GLS were observed in several participants, while two met criteria for mild asymptomatic cancer therapy–related cardiac dysfunction (CTRCD). However, changes in VO_2_ peak did not consistently correlate with subclinical cardiac dysfunction assessed by GLS. Notably, participants generally reported better HRQoL and lower fatigue levels. These findings illustrate the lack of a consistent parallel between functional capacity, subclinical cardiac changes, and PROs.

In terms of behavioural support, a diverse array of support strategies was utilized, reflecting variability in implementation protocols across trials. One notable effect was increased adherence through individualized goal-setting and strategies to manage barriers related to functional status and treatment symptoms.

Costa et al. (2025) [34] reported that physical activity and behavioural support may help preserve both cardiac and physical function during chemotherapy. Although many participants reported persistent fatigue, which negatively impacted HRQoL, there were positive signs that physical activity might improve it. However, given the pilot nature and limited sample size of the study, these findings remain preliminary and do not establish that changes in HRQoL were attributable to cardioprotection.

Spector et al. (2014) [40] used a home-based intervention with weekly motivational counselling, reinforcing adherence. Participants showed improvements in cardiorespiratory fitness, functional movement, and HRQoL scores, although statistical significance was not reached. These findings support the potential value of exercise and behavioural support for functional outcomes and PROs, without demonstrating a cardioprotective mechanism.

Griffith et al. (2009) [41] combined behavioural support with a home-based brisk-walking program and found positive trends in cardiorespiratory performance. Interestingly, prostate cancer patients showed significantly better maintenance or improvement in VO_2_ peak, compared to other cancer types. This study also highlighted the potential of physical activity in alleviating pain. As the intervention primarily targeted physical function and symptoms rather than cardiac injury, these findings are more appropriately interpreted as evidence for the supportive role of exercise rather than cardioprotection.

**Section D. Supportive-care studies**

Koop et al. (2022) [42] demonstrated that CTRCD can impose an additional burden on patients beyond the cancer diagnosis itself, affecting physical, psychosocial, and social domains of HRQoL. Through semi-structured interviews, participants frequently described fatigue as “all-consuming” and difficult to distinguish from the broader effects of cancer treatment. The interpretation of cardiovascular symptoms was also identified as a source of anxiety, adding to the psychological burden associated with cancer treatment. Patients reported difficulties in understanding and accepting their functional limitations, which were further intensified by CTRCD. Many participants felt that CTRCD was insufficiently recognized within the healthcare system, often due to limited acknowledgement of symptoms and inadequate professional communication, which negatively affected their healthcare experiences and ability to understand their condition. As a result, patients emphasized the need for individualized follow-up strategies and multidisciplinary coordination between oncology and cardiology specialists.

## 4. Discussion

This review aimed to address two complementary objectives:

A. to examine the evidence linking HRQoL with (i) documented cardiac injury or cardiovascular disease, (ii) imaging- or biomarker-detected cardiovascular abnormalities, (iii) interventions aimed at preventing or treating cardiotoxicity, and (iv) general supportive interventions that improve HRQoL without demonstrated cardioprotection;

B. to synthesize the available evidence according to four major study domains: diagnostic and cardiovascular assessment, pharmacological and cardioprotective interventions, exercise-based interventions, and qualitative or supportive-care approaches.

A. The findings are discussed below in relation to the four research questions addressed by the included studies.

RQ 1: Clinically manifest cardiovascular disease and HRQoL

The evidence addressing RQ1 provides the clearest patient-centred signal within the cardiovascular domains examined in this review: once cardiovascular disease or cardiac dysfunction becomes clinically manifest, its association with impaired HRQoL becomes more apparent. This is clinically plausible because established cardiovascular disease can directly affect functional capacity, exercise tolerance, fatigue, dyspnoea, and the ability to perform everyday activities. Supporting this interpretation, Vasbinder et al. [46] recently showed that incident cardiovascular disease after breast cancer was associated with poorer physical and mental QoL, reinforcing the relevance of cardiovascular disease to long-term survivorship outcomes.

Importantly, this relationship should not be interpreted as a purely cardiovascular effect. Cancer survivors with established cardiac disease often experience overlapping treatment-related symptoms, comorbidities, psychological distress, and physical deconditioning, all of which may contribute to poorer HRQoL. The 2025 Heart Failure Society of America scientific statement by Weisfelner Bloom et al. [47] emphasizes the complex intersection between cardiovascular and oncological disease and supports a multidisciplinary approach to cardiovascular care in patients with active cancer and cancer survivors. Similarly, Keramida et al. [48] highlighted the complex clinical presentation of heart failure with preserved ejection fraction in patients with cancer and survivors, in whom symptoms and functional limitations may be substantial despite the preservation of conventional measures of left ventricular systolic function. Together, these observations reinforce the multidimensional nature of HRQoL in patients with concomitant cardiovascular and oncological disease.

The small number of studies addressing RQ1 may itself reflect an important feature of the field. Contemporary cardio-oncology has increasingly shifted toward preventing or detecting cardiovascular injury before overt dysfunction develops. The recent consensus statement by Rakisheva et al. [49] illustrates this prevention-oriented direction, emphasizing risk stratification, early detection, and prevention of CTRCD. Consequently, the literature is richer in studies of subclinical abnormalities and cardiotoxicity prevention than in studies directly examining how established cardiovascular disease affects the patients’ perceived health. This suggests an important evidence gap in patient-centred survivorship research: the clinical relevance of overt cardiovascular disease is increasingly recognized, but its longitudinal relationship with HRQoL remains less systematically characterized.

RQ 2: Subclinical cardiovascular abnormalities and subsequent HRQoL

The limited evidence directly addressing RQ2 highlights an important gap in contemporary cardio-oncology: the clinical relevance of subclinical cardiovascular abnormalities from the patient’s perspective. Contemporary cardio-oncology has increasingly moved toward detecting myocardial injury before overt cardiac dysfunction develops, using biomarkers, global longitudinal strain, and advanced imaging. Sartorio et al. and Qiu et al. [50,51] have highlighted the role of strain-based imaging, particularly GLS and more comprehensive speckle-tracking parameters, in detecting early myocardial abnormalities before conventional measures of cardiac function become abnormal. This approach is valuable for prevention, but the detection of an abnormal cardiac parameter does not necessarily imply an immediate deterioration in HRQoL.

This distinction may help explain why the association between subclinical cardiovascular abnormalities and HRQoL remains poorly characterized. Biomarker elevation or strain impairment may precede symptoms by months, while HRQoL is simultaneously influenced by cancer-related symptoms, treatment toxicity, fatigue, psychological distress, and physical deconditioning. Thus, the cardiovascular signal detected by imaging or biomarkers and the patients’ perceived health status may not change synchronously. Importantly, early cardiac abnormalities may also be reversible, further complicating the assumption that their detection should directly translate into a measurable HRQoL decline. Qiu et al. [51] demonstrated the dynamic nature of strain abnormalities during and after anthracycline treatment, while Camilli et al. [52] identified abnormal strain-derived myocardial work indices in cancer survivors without reported cardiological symptoms, further illustrating the potential presence of subclinical cardiovascular abnormalities despite the absence of clinically perceived cardiac symptoms.

The scarcity of RQ2 evidence therefore represents more than a numerical gap in the literature. It reflects a broader gap between early biological detection and patient-centred clinical relevance. Future longitudinal studies should integrate serial cardiovascular biomarkers and imaging with repeated HRQoL assessments to determine whether specific subclinical abnormalities precede, accompany, or remain clinically dissociated from deterioration in patient-reported health.

RQ 3: Cardioprotective interventions and HRQoL

The predominance of RQ3 studies reflects an important evolution in cardio-oncology: the field is increasingly moving beyond the detection of cardiotoxicity toward interventions designed to prevent, attenuate, or manage cardiovascular consequences while preserving patients’ QoL. Recent evidence supports this broader preventive paradigm, with pharmacological and non-pharmacological strategies increasingly evaluated across the cancer treatment continuum. Migliari et al. [53] emphasized that contemporary cardioprotection encompasses multiple therapeutic approaches, although the magnitude and clinical relevance of their cardiovascular benefit remain variable.

At the same time, RQ3 represents the most methodologically heterogeneous evidence domain in this review. Pharmacological cardioprotection and exercise-based interventions may both be considered within a cardiotoxicity-prevention framework, but they do not necessarily improve HRQoL through the same mechanisms. Recent evidence illustrates this distinction particularly well for exercise: Zhang et al. [54] found improvements in cardiac function and GLS with exercise-based interventions, but emphasized the low certainty of evidence regarding prevention of cancer therapy-related cardiac dysfunction. Conversely, Marmol-Perez et al. [55] demonstrated that exercise can improve HRQoL after cancer independently of whether a cardioprotective mechanism has been established. Thus, an improvement in HRQoL following an intervention should be interpreted as a patient-centred benefit rather than evidence that cardioprotection mediated this effect.

This distinction is important when interpreting the findings of RQ3. The growing emphasis on cardio-oncology rehabilitation reflects an increasingly patient-centred model of care. However, recent expert recommendations from the ICOS-CORE working group also highlight substantial methodological limitations in the existing evidence and call for standardized cardiovascular and patient-centred outcomes [56]. Accordingly, future trials should prospectively evaluate both dimensions and, where possible, examine whether preservation of HRQoL is associated with—or independent of—the intervention’s demonstrable cardiovascular effects.

RQ4: Supportive interventions and HRQoL without demonstrated cardioprotection

RQ4 underscores an important consideration in the interpretation of PROs in cardio-oncology: clinical benefit does not necessarily depend on demonstrating a cardioprotective effect. Supportive interventions may enhance HRQoL through direct improvements in physical functioning, fatigue, symptom burden, psychological well-being, and treatment-related burden, rather than through measurable changes in cardiac structure or function. This distinction is particularly relevant to exercise, which is increasingly recognized as an integral component of supportive cancer care. Consistent with this broader perspective, studies such as Scott et al. [57] have demonstrated significant improvements in cardiorespiratory fitness following exercise interventions in patients with cancer, highlighting benefits that extend beyond potential cardiovascular protection.

The distinction between benefit and mechanism is therefore critical. An improvement in HRQoL following exercise or supportive care should be interpreted as evidence of patient benefit, but not automatically as evidence of cardioprotection. Recent work by Cormie et al. [58] emphasizes that exercise in oncology has multiple therapeutic targets, including physical function, fatigue, psychological well-being, and quality of life, while cardiovascular effects represent only one potential pathway through which exercise may benefit patients with cancer. Similarly, Schmitz et al. [59] have highlighted the broader role of exercise as an integral component of supportive cancer care rather than exclusively as a strategy for preventing treatment-related cardiovascular toxicity.

The relatively small representation of RQ4 in the present review may reflect the specific focus of the literature retrieved rather than the limited clinical importance of supportive care. Much of the cardio-oncology literature is structured around cardiotoxicity prevention, whereas exercise and supportive-care research is more commonly framed around rehabilitation, survivorship, fatigue, and functional recovery. Consequently, these evidence streams may remain conceptually separated, despite addressing overlapping PROs. Recognizing this distinction prevents over-attribution of HRQoL benefits to cardioprotection and supports a more accurate interpretation of the multifactorial determinants of QoL in patients with cancer.

Across the four research questions, the observed relationships should be interpreted as associations rather than evidence of causality, particularly in observational studies. HRQoL may also be influenced by concurrent cancer- and treatment-related factors, including fatigue, psychological distress, functional impairment, and other systemic or clinical conditions that may overlap with both cardiovascular status and treatment-related symptoms.

B. The findings are further considered below across four complementary study domains.

Contemporary literature indicates that cancer therapy–related cardiotoxicity encompasses a broad spectrum of cardiovascular manifestations, including hypertension, arrhythmias, ischemic events, and inflammatory syndromes; however, the most clinically relevant and extensively investigated manifestation remains CTRCD, which may progress to overt heart failure [11,60,61].

From a clinical perspective, heart failure represents the main pathway through which cardiotoxicity affects patients’ HRQoL. The symptoms most frequently associated with this progression, particularly fatigue and dyspnea, substantially impair physical functioning and daily activities, although their impact may extend beyond the physical domain to broader aspects of patient well-being [60,62]. The presence of these symptoms reflects a clinically meaningful burden, as similar patterns of functional limitation have been consistently reported across heart failure of different etiologies, highlighting their disabling nature despite their non-specific presentation [62].

From a therapeutic perspective, several cardioprotective strategies, including renin–angiotensin system inhibitors and beta-blockers have demonstrated effects on surrogate measures of cardiac dysfunction, while dexrazoxane is recognized in the 2022 ESC Cardio-Oncology Guidelines as a cardioprotective strategy that may be considered in selected patients receiving anthracyclines who are at high or very high risk of cancer therapy-related cardiac dysfunction (CTRCD) or who require high cumulative anthracycline exposure [11]. Emerging cardioprotective interventions, including statins and sodium-glucose cotransporter-2 (SGLT2) inhibitors, have shown encouraging preliminary evidence for attenuating cancer therapy-related cardiac injury and limiting the progression toward left ventricular dysfunction, with statins demonstrating potential for reducing anthracycline-associated left ventricular dysfunction in high-risk populations and SGLT2 inhibitors emerging as promising agents based on preclinical and early clinical evidence of cardioprotective effects [63,64,65,66]. Nevertheless, despite these favourable effects on cardiovascular surrogate endpoints, current evidence has not yet demonstrated consistent improvements in HRQoL or other PROs.

From a paraclinical perspective, early identification of cardiotoxicity relies on a structured surveillance strategy aimed at detecting subclinical cardiac injury before the development of irreversible dysfunction.

Current recommendations advocate baseline cardiovascular assessment before the initiation of potentially cardiotoxic therapies, followed by longitudinal monitoring tailored to the specific cancer therapy and patient risk, incorporating electrocardiography (ECG), transthoracic echocardiography (TTE) with assessment of LVEF and GLS, serial cardiac biomarkers, and, when indicated, advanced cardiovascular imaging modalities [67,68,69,70]. This stepwise, multidisciplinary approach facilitates early recognition of cardiac abnormalities and enables prompt intervention, thereby reducing the risk of progression from subclinical myocardial injury to clinically overt heart failure [67,68,69,71].

At the practical level, current ESC recommendations emphasize baseline cardiovascular assessment before initiating potentially cardiotoxic therapies, incorporating clinical evaluation, formal cardiovascular risk stratification according to the 2022 ESC Cardio-Oncology Guidelines, including the HFA-ICOS risk assessment framework [11,67], ECG, and TTE, including assessment of LVEF and GLS [11,67,68]. The 2022 ESC Guidelines advocate a risk-adapted surveillance strategy, whereby the intensity and frequency of cardiovascular surveillance are tailored to the patient’s baseline cardiovascular risk, the specific anticancer therapy, and its expected cardiotoxicity profile, with LVEF and GLS assessment by echocardiography and longitudinal measurement of cardiac biomarkers when indicated. During treatment, longitudinal monitoring with cardiac biomarkers, particularly troponins and natriuretic peptides, alongside repeated imaging assessments using echocardiography, strain analysis, and, when appropriate, cardiac magnetic resonance imaging, facilitates earlier detection of myocardial injury [11,68,69]. Serial assessment of LVEF and GLS, complemented by cardiac biomarkers when indicated, may facilitate the detection of subclinical cardiac dysfunction before overt heart failure develops [11]. It is important to note that although these paraclinical tools provide essential objective markers for the early identification and management of cardiotoxicity, they do not necessarily capture the patient-centred consequences of treatment. The findings of the present review therefore support a complementary approach in which objective cardiovascular surveillance is considered alongside HRQoL and other PROs, particularly when early or subclinical cardiovascular abnormalities are detected. This highlights the need to better define their relationship with HRQoL outcomes.

CMR is increasingly recognized as a highly sensitive modality for myocardial tissue characterization and the detection of early subclinical myocardial injury, particularly in high-risk or equivocal cases, while TTE remains the first-line imaging technique recommended by current guidelines; the two modalities therefore play complementary roles within a multimodality diagnostic approach [67,68,69,70]. The current role of CMR remains primarily centred on biological and structural assessment. Whether CMR-derived abnormalities reflect or predict patients’ symptoms, functional limitations, and HRQoL impairment requires further investigation. In the current review, only a single study was identified that specifically investigated the relationship between CMR-detected cardiac changes and deterioration in HRQoL. This highlights a critical evidence gap between advanced imaging-based detection of cardiotoxicity and its translation into meaningful patient-centred outcomes.

Our review also examined chemotherapy strategies intended to mitigate cardiotoxicity and their potential effects on HRQoL in patients with cancer. Many of the included studies focused on anthracyclines, which are well-known for their cytotoxic effects. This reflects the prevalent use of anthracyclines in the treatment of breast cancer, the primary cancer type studied. The second most common therapy was trastuzumab, a targeted therapy often used in combination with anthracyclines to enhance breast cancer treatment outcomes.

Given the well-established cardiotoxicity of chemotherapy, it might be expected that avoiding concomitant administration of multiple cardiotoxic agents would reduce cumulative cardiac injury. However, our findings suggest that a concomitant regimen of anthracyclines and taxanes may not significantly affect either cardiac function or HRQoL. Similarly, trastuzumab, when added to anthracyclines— including in patients with mild cardiotoxicity —can generally be continued without significant detriment, provided that appropriate cardioprotective treatments are in place. Regarding cumulative chemotherapy exposure, our findings align with existing research, suggesting the minimal role of cumulative chemotherapy doses in severe cardiac impairment [72].

Beyond pharmacological cardioprotection, emerging evidence suggests a potential role for non-pharmacological approaches during cardiotoxic cancer therapy. Ignatenko et al. [73] investigated intermittent normobaric hypoxic therapy as an adjunct to anthracycline-based neoadjuvant chemotherapy in women with invasive breast cancer, reporting favourable findings across selected cardiovascular and patient-reported outcomes, including domains of the SF-36 questionnaire. This strategy is of particular interest because it extends the concept of cardioprotection beyond conventional pharmacological interventions and highlights the potential contribution of non-pharmacological strategies to cardiovascular protection during cancer treatment. Although further evidence is required to establish the efficacy and clinical applicability of these interventions, they may represent an additional avenue for investigation in the evolving field of cardio-oncology.

The role of physical activity as a cardioprotective strategy in cardio-oncology has been extensively studied in both human and animal models, with a particular focus on anthracyclines [74,75]. The role of physical activity as a cardioprotective strategy in cardio-oncology has been extensively studied in both human and animal models, with a particular focus on anthracyclines [74,75]. While ten studies assessed various exercise regimens and provided some evidence suggesting favourable associations between physical activity, cardiorespiratory fitness, and HRQoL, the findings were heterogeneous and should be interpreted in light of the substantial methodological diversity across exercise regimens, HRQoL assessment tools, and methods used to assess cardiac function, which limits consistent and direct comparisons across studies. Importantly, favorable changes in HRQoL and cardiorespiratory fitness may reflect functional benefits of exercise without necessarily indicating a cardioprotective effect, which requires objective assessment of cardiovascular outcomes.

Regarding psychosocial and behavioural support, its role extends beyond promoting treatment adherence and healthy lifestyle: it represents a fundamental component of comprehensive cancer care and has been consistently associated with better coping, reduced emotional distress, and improved HRQoL. Within the field of psycho-oncology, interventions such as patient education, psychological counselling, motivational interviewing, goal setting, stress-management strategies, and structured exercise programs are recognized as effective approaches for addressing both the physical and emotional consequences of cancer and its treatment [76,77].

Psychosocial support may additionally enhance the benefits of exercise-based interventions when delivered as part of a broader care framework, functioning both as an independent therapeutic resource and as a complementary component to other strategies, thereby helping patients maintain HRQoL despite the cardiotoxic burden of chemotherapy. These observations reinforce the need for multidisciplinary cardio-oncology programs that integrate psychological support alongside cardiovascular prevention and rehabilitation strategies [56,78].

Regarding L-arginine, its cardioprotective effects are primarily mediated through nitric oxide (NO)-dependent pathways, promoting vasodilation, improving coronary perfusion, and modulating endothelial and apoptotic signalling [79,80]. Clinical evidence in patients receiving anthracycline-based chemotherapy suggests that L-arginine may improve endothelial function and reduce early markers of anthracycline-related cardiotoxicity [31]. Furthermore, clinical evidence suggests that L-arginine supplementation during anthracycline-based induction chemotherapy may be associated with improved HRQoL, particularly in patients with concomitant ischemic heart disease, supporting a potential link between cardioprotective metabolic modulation and patient-reported outcomes in the cardio-oncology setting [81]. Although these findings are encouraging, they are derived from limited clinical evidence and should therefore be interpreted with caution. Further well-designed clinical studies are needed to determine whether nutritional interventions, including L-arginine supplementation, provide clinically meaningful cardioprotective benefits and improve HRQoL in patients receiving potentially cardiotoxic cancer therapies [31,81].

The final strategy identified in our review involved the use of semi-structured interviews to assess patient experiences [42]. This qualitative approach highlighted the importance of communication skills among healthcare providers, emphasizing the need for effective communication regarding the challenges associated with cardiac impairment following chemotherapy. This study offers valuable insights into the patients’ perspectives and could guide future research efforts, particularly with regard to improving medical awareness, fostering collaboration among specialties, and promoting personalized treatment plans in cardio-oncology. These findings are consistent with the multidisciplinary approach advocated by the 2022 ESC Cardio-Oncology Guidelines, which emphasize coordinated cardiovascular and oncological assessment and management according to the patient’s cardiovascular risk and cancer treatment. In this context, multidisciplinary cardio-oncology care may facilitate individualized decisions regarding cardiovascular surveillance, prevention, and management, while ensuring that patient-reported symptoms and HRQoL are incorporated into clinical decision-making [11].

Although digital health technologies and wearable devices are increasingly recognized as promising tools for continuous monitoring of cardiovascular status, physical activity, and symptom burden in patients undergoing cancer therapy, evidence supporting their impact on patient-reported outcomes, including HRQoL, remains limited to date [82].

In terms of preserving HRQoL, existing evidence suggests that specific chemotherapy strategies, structured exercise, and individualized nutritional guidance may have a moderate effect on HRQoL impairment. However, firm conclusions cannot yet be drawn because of the lack of large-scale, well-controlled prospective studies and methodological inconsistencies across available evidence. More robust research is therefore needed to determine the most effective strategies for protecting cardiac health and optimizing HRQoL. In this context, digital health technologies and wearable devices represent a potential complementary future direction for continuous monitoring and functional assessment in cancer patients, although their clinical impact on HRQoL remains insufficiently defined.

## 5. Limitations

Although a structured literature search and predefined eligibility criteria were used to enhance the transparency of study identification, the present work was intentionally designed as a narrative review to accommodate the considerable methodological and clinical heterogeneity of the available evidence. Consequently, the findings should be interpreted as an integrative synthesis rather than an exhaustive assessment of all published studies.

The included studies varied substantially in patient populations, cancer types and treatment regimens, cardiovascular assessments, and HRQoL instruments, limiting direct cross-study comparisons. Even if chemotherapy was a required component of treatment in the eligibility criteria, several studies involved multimodal treatment approaches, including targeted therapies, radiotherapy, hormonal therapy, or other concomitant treatments. Consequently, the cardiovascular and HRQoL outcomes reported in these studies should be interpreted in the context of the overall treatment regimen rather than being considered attributable to chemotherapy alone. The available evidence comprised heterogeneous study designs, including observational studies, with many investigations limited by small sample sizes and consequently reduced statistical power. Accordingly, findings from smaller descriptive studies were interpreted primarily as supportive and hypothesis-generating rather than definitive.

Additional limitations include the potential for language and publication bias, as well as selection bias related to study identification and eligibility criteria. No formal risk-of-bias or quality assessment was performed, consistent with the narrative design of the review. Differences in the definitions and assessment of cardiotoxicity, cardiovascular dysfunction, and HRQoL, together with the indirectness of some included studies, may have influenced the consistency of the findings. Finally, although several studies reported parallel changes in cardiovascular measures and HRQoL, the available evidence generally does not establish that changes in HRQoL were directly caused by cardiotoxicity, particularly given the multiple clinical, functional, and psychosocial determinants of patient-reported health.

## 6. Conclusions

Our study supports a clinically relevant relationship between cardiovascular health and HRQoL across cancer treatment and survivorship, although its strength and interpretation vary according to the degree of cardiovascular involvement and the intervention examined. Established cardiovascular disease or cardiac dysfunction shows the clearest association with impaired HRQoL, particularly in physical domains, whereas the patient-reported impact of subclinical cardiovascular abnormalities remains less well characterized. Early cardiovascular assessment may facilitate the timely identification of subclinical abnormalities, but the available evidence does not demonstrate that imaging-based detection itself improves HRQoL. Pharmacological cardioprotective and exercise-based interventions have been more extensively studied, but improvements in HRQoL cannot be attributed to cardioprotection without objective evidence of cardiovascular benefit. Supportive interventions may also improve HRQoL through functional, symptomatic, or psychosocial pathways, independent of cardiovascular protection. These findings should be considered in the context of heterogeneity across study populations, endpoints, HRQoL measures and study designs, with many studies based on small observational or feasibility cohorts. Larger prospective longitudinal studies using standardized HRQoL measures as predefined primary endpoints would help clarify the clinical impact of early cardiovascular injury and determine whether effective cardiovascular protection can translate into better patient-reported health.

## Figures and Tables

**Table 1:** Characteristics of the included studies.

**A. Diagnostic Studies**										
Author, Country, Publication Year	Study Characteristics	Cancer Type/ Chemotherapy	Intervention/ Exposure	Cardiac Endpoint	Cardiotoxicity Definition	HRQoL Instruments, HRQoL Endpoint (Type)	Follow-Up	Main Findings	Adjusted Analysis	Study Focus
Buziashvili et al., Russia (2024) [20]	Observational cohort study (*n* = 72)	Breast cancer PH-FECH ± trastuzumab	Neoadjuvant chemotherapy; continuous cardiac monitoring	Continuous cardiovascular monitoring; echocardiographic assessment including LVEF	No explicit definition; subclinical cardiotoxicity monitored, specific parameters not reported	SF-36, EQ-5D-5L, Karnofsky, ECOG change in QoL after NAC (primary)	Baseline, after cycle 4, post-NAC.	QoL and functional status deteriorated during NAC, particularly in physical functioning and symptom domains	No multivariable adjustment; comparisons, ORs and relative risks	RQ 4
Drafts et al., USA (2013) [21]	Observational cohort study (*n* = 53)	Breast cancer, leukaemia, lymphoma Anthracycline-based (50–375 mg/m^2^ doxorubicin-equiv.)	Anthracycline-based chemotherapy	LVEF, LV strain, LV volumes, PWV, CMR-LGE, BNP, TnI	No formal definition; subclinical cardiac injury assessed by TnI	MLHFQ change over time (secondary)	Baseline, 1 month, 3 months, 6 months	LVEF, LV strain and PWV worsened, while QOL deteriorated over 6 months; changes persisted after adjustment	Age, gender, race, dose, administration, CV comorbidities, cancer diagnosis	RQ 2
Geršak et al., Germany (2021) [22]	Prospective observational cohort (*n* = 59)	Breast cancer: (EC-P ± dose-dense) ± trastuzumab; NHL: (R-CHOP) HL: anthracycline-based combinations ± radiation	Chemotherapy; serial cardiovascular assessment	Clinical cardiotoxicity; cardioprotective therapy indication; LVEF, GLS and fast-SENC CMR strain parameters	Clinical: LVEF ↓ >10% to <53% + HF symptoms/biomarkers; subclinical: asymptomatic LVEF ↓>15%, GLS ↓>15%, or abnormal biomarkers	EQ-5D, SF-36 (HRQoL decline >5%) (secondary)	Baseline, 1 month, 3 months, 6 months, 1 year	Clinical indicators of cardiac dysfunction were significantly associated with poorer reported QoL. Among imaging measures, only MyoHealth segmental myocardial strain showed a consistent association with QoL decline (Fast-SENC CMR)	No multivariable adjustment; survival analysis	RQ 2
Lubberts et al. Netherlands (2023) [23]	Multicenter cohort study (*n* = 4748 TC survivors; 272 developed CVD)	Testicular cancer All TC: surgical; Early-stage seminoma: orchidectomy ± radiotherapy (26–35 Gy); Stage II–IV non/seminoma: cisplatin-based chemotherapy (BEP); Older regimens: cisplatin, vinblastine, bleomycin	Previous testicular cancer treatment; long-term assessment of CVD risk and QoL	Cardiac endpoint: incident CVD after TC treatment	No formal definition reported; documented CVD: CAD, MI or HF	SF-36 (secondary)	16.1 years	Those with CVD reported poorer QoL (physical domains). Cisplatin-based chemotherapy was associated with increased CVD risk Obesity, smoking, Raynaud’s phenomenon, dyslipidaemia, and family history of CVD were also independently associated with CVD risk	Linear regression; adjusted for age, time since TC diagnosis, and time since CVD	RQ 1
**B. Cardioprotective Pharmacological Studies**										
Author Country, Publication Year	Study Characteristics	Cancer Type/ Chemotherapy	Intervention/ Exposure	Cardiac Endpoint	Cardiotoxicity Definition	HRQoL Instruments, HRQoL Endpoint (Type)	Follow-Up	Main Findings	Adjusted Analysis	Study Focus
Huang et al., China (2022) [24]	Retrospective comparative study (*n* = 86)	Osteosarcoma Anthracycline (doxorubicin conventional or liposomal) + ifosfamide	Ifosfamide (1.5 g/m^2^ i.v.) + liposomal doxorubicin (50 mg/m^2^ i.v.), q3 weeks × 6 cycles	Cardiac toxicity recorded as an adverse reaction; 8/40 (20.0%) in the conventional doxorubicin group vs. 2/46 (4.35%) in the liposomal doxorubicin group, p = 0.024	Not defined; diagnostic criteria and severity grading not reported.	EORTC QLQ-C30, ECOG, Karnovsky. (endpoint status not explicitly specified)	Treatment period: 6 courses. HRQoL: before and after therapy. Overall survival: 2-year follow-up, with monthly contact	Liposomal doxorubicin was associated with lower cardiotoxicity and higher QLQ-C30 scores than conventional doxorubicin	No adjusted analysis reported. Comparisons were performed using χ^2^ tests, independent-samples t tests, paired t tests, Kaplan–Meier analysis and log-rank testing	RQ 3
Sideras et al., USA (2022) [25]	Randomized controlled trial (*n* = 45)	Breast cancer Anthracyclines ± taxanes in neoadjuvant, adjuvant, or metastatic setting	Group A: pixantrone i.v. 180 mg/m^2^ q3 weeks Group B: pixantrone i.v. 85 mg/m^2^ on days 1, 8, 15 q4 weeks	Cardiotoxicity: decrease in ejection fraction and cardiac adverse events/cardiotoxicity	CTCAE-graded EF decrease; specific EF threshold or operational definition not reported	LASA-6 (secondary)	During treatment; median number of treatment cycles was 3, subsequently followed until death; median OS was 16.8 months overall	Limited cardiotoxicity, with EF decline reported in 4% of patients (mean decline, 2%). No clear evidence that pixantrone improved QoL or prevented QoL deterioration	No adjusted analysis reported	RQ 3
Conte et al., Italy/Spain (2004) [26]	Randomized controlled trial (*n* = 202)	Breast cancer Anthracycline (≤360 mg/m^2^ epirubicin or ≤240 mg/m^2^ doxorubicin)	Arm A (Concomitant): epirubicin 90 mg/m^2^ + paclitaxel 200 mg/m^2^ q3 weeks × 8 cycles Arm B (Sequential): epirubicin 120 mg/m^2^ q3 weeks × 4 cycles → paclitaxel 250 mg/m^2^ (3-h infusion) q3 weeks × 4 cycles	CHF; LVEF decline	Grade ≥ II CHF (NYHA) or ≥ 20% decrease in LVEF	QLQ-C30 (secondary)	Baseline, QoL: cycles 2, 4, 8 Cardiac: every 2 cycles. Median 8 cycles administered in both arms	No consistent differences in clinically relevant cardiotoxicity or QoL were observed between treatment arms. More grade II cardiotoxicity/heart failure events occurred with concomitant treatment than with sequential treatment, but overall cardiotoxicity did not differ significantly between the two strategies	Adjusted mixed-effects analysis for HRQoL; adjusted for baseline QOL and dropout-related covariates; no adjusted cardiac analysis	RQ 3
Suntheralingam et al. Canada (2024) [27]	Retrospective matched cohort study (*n* = 14)	Breast cancer Sequential anthracyclines followed by trastuzumab (± radiotherapy)	Continuation vs. temporary interruption of trastuzumab in patients with mild CTRCD receiving HF therapy (β-blockers and/or ACEi/ARB)	Primary: CMR-measured LVEF <40% after trastuzumab. Secondary: LVEF, LV volumes, myocardial edema (T2), fibrosis (T1/ECV), clinical HF	Mild CTRCD: ≥10% decline in LVEF to < 55%, with nadir > 40%, measured by CMR	MLHFQ, EQ-5D-3L (secondary)	CMR at baseline, every 3 months during therapy, post-trastuzumab, and 2-year follow-up; median follow-up 2.5 years	Continuing Trastuzumab in patients with mild cardiotoxicity was not associated with significant worsening of cardiac function or QoL impairment compared with treatment interruption	Fisher exact/Mann–Whitney tests for between-group comparisons; GEE for longitudinal outcomes; no multivariable adjusted analysis	RQ 3
Ganz et al., USA (2017) [28]	Randomized controlled trial (*n* = 441)	Breast cancer Doxorubicin + cyclophosphamide (AC) → paclitaxel	Trastuzumab i.v. 4 mg/kg loading dose with first paclitaxel → 2 mg/kg weekly × 52 weeks	Primary: significant LVEF decline at LTF: ≥10% absolute decline from baseline to LVEF < 50% Secondary: LVEF < 40% at LTF among those with LVEF > 50% at 18 months; cardiac symptoms/comorbidities and functional status	≥10% absolute LVEF decline from baseline to < 50% at LTF	MOS SF-36, DASI, a comorbidity questionnaire [29], a cardiac symptom checklist [30] (secondary)	Median 8.8 years from randomiza-tion; LTF assessment performed 5–10 years after randomiza-tion (median questionnaire completion 8.7 years)	In patients without pre-existing heart disease, adding Trastuzumab to standard chemotherapy was not associated with long-term cardiac dysfunction or reduced QoL. DASI scores were more strongly related to age and comorbidities than to Trastuzumab exposure	Multivariable logistic regression for low DASI (< 43); no adjusted analysis for LVEF endpoint	RQ 3
Skrypnyk et al. Ukraine (2019) [31]	Prospective comparative study; (*n* = 147)	Acute leukaemia AML: “7 + 3”/”5 + 2” regimen ALL: BFM regimen	L-arginine hydrochloride 4.2% 100 mL i.v. on the day before and on days of anthracycline administration > oral L-arginine aspartate (5 mL), 3 times daily × 30 days	Anthracycline-induced myocardial injury/ cardiotoxicity	No explicit clinical cardiotoxicity definition reported	SF-36 (secondary)	Baseline, After induction chemo-therapy	L-arginine supplementation during anthracycline-based induction chemotherapy was associated with reduced anthracycline-related myocardial injury and improved QoL, particularly among patients with comorbid ischemic heart disease	No multivariable adjusted analysis reported	RQ 3
**C. Exercise Studies**										
Author Country, Publication Year	Study Characteristics	Cancer type/ Chemotherapy	Intervention/ Exposure	Cardiac Endpoint	Cardiotoxicity Definition	HRQoL Instruments, HRQoL Endpoint (Type)	Follow-Up	Main Findings	Adjusted Analysis	Study Focus
Heinze-Milne et al. Canada (2021) [32]	Prospective feasibility study (*n* = 10)	Breast cancer, hematological cancer Anthracycline-based (doxorubicin/ epirubicin/ daunorubicin)	Physical activity	Cardiac function and biomarkers of cardiotoxicity	No explicit operational definition reported	FACT (FACT-G, FACT-Lym, FACT-B), FACIT-F, IPAQ (secondary)	Baseline and post-intervention (12 weeks), during anthracycline treatment	Exercise was feasible and safe during anthracycline chemotherapy, but no significant improvement in QoL, fitness, fatigue, or biomarkers was demonstrated	No multivariable adjusted analysis reported	RQ 3
Hughes et al., USA (2011) [33]	Case reports (*n* = 2)	HL, Leukaemia Anthracycline-based (initiated 13 and 5 years prior to investigation)	Physical activity	LVEF, BNP, VO_2_ peak; HF symptoms/exercise tolerance	Pre-existing chemotherapy-related HF, NYHA class II–III; no separate operational definition of cardiotoxicity reported	SF-36, MDASI—HF, 7-DPARQ (secondary)	16 weeks	A 16-week exercise intervention to cancer survivors with chemotherapy-induced HF resulted in improvements in cardiovascular function and QoL	Descriptive case-level comparisons only	RQ 3
Costa et al. USA (2025) [34]	Randomized controlled pilot feasibility trial (*n* = 18)	NHL, HL Anthracycline-based or other cancer therapies potentially affecting CV function (immunotherapy or radiotherapy)	Physical activity	Exploratory: MRI-derived resting/ exercise LVEF, LV stroke volume and cardiac output	Cardiac function was assessed by MRI; resting LVEF <50% occurred in some participants but was not used as a formal cardiotoxicity definition	FACT-G, FACT-Lym FACIT–F (exploratory outcome)	Baseline, 3 and 6 months, during active cancer treatment	The tailored physical activity intervention was feasible, well tolerated and associated with improved exercise capacity and preservation of CV function during treatment. Preliminary findings suggested a potential HRQoL benefit	Descriptive comparisons of means/SD across groups and timepoints; no multivariable adjusted analysis reported	RQ 3
Murray et al. Australia (2023) [35]	Case series (*n* = 5)	Breast cancer Dose-dense anthracycline-based	Physical activity	GLS; additional cardiac parameters included LVEF, RV free-wall strain, EDV, ESV, resting HR, stroke volume and cardiac output	Mild asymptomatic CTRCD: LVEF ≥ 50% and relative GLS decline ≥ 15% from baseline. No participant met moderate/severe CTRCD criteria	QLQ-C30 (primary) fatigue, QLQ-FA12 (secondary)	Baseline; 4 weeks; 8 weeks (completion of DDAC); 20 weeks (3 months after DDAC completion)	Aerobic exercise was associated with favourable QoL and fatigue outcomes; CV responses varied across participants	No adjusted analysis; individual participant data were presented descriptively	RQ 3
Schneider et al. Switzerland (2024) [36]	Observational (*n* = 262)	Breast cancer, lymphoma, blood cancer, other Anthracycline-based chemotherapy +/− other cardiotoxic treatment; non-anthracycline cardiotoxic treatment (e.g., trastuzumab, left-sided radiotherapy, fluorouracil or cyclophosphamide); non-cardiotoxic treatment	Physical activity	No direct cardiac endpoint assessed; VO_2_ peak was the primary outcome	Not formally defined/assessed; cardiotoxicity was based on treatment classification	FACT-G, FACIT-F (secondary)	Before and after exercise training; timing classified as during vs. after chemo-therapy	VO_2_ peak gains were attenuated during anthracycline therapy but greater when exercise was initiated after treatment; no timing-related differences were observed with non-anthracycline therapies	VO_2_ peak: robust linear model adjusted for therapy type, exercise timing, age, training impulse, and baseline VO_2_ peak	RQ 3
Peck et al. Canada (2022) [37]	Observational (*n* = 88)	Breast cancer Sequential anthracycline → trastuzumab	Physical activity	LVEF, GLS, E/A, E/e′; diastolic dysfunction; CTRCD	CMR-defined CTRCD: ≥ 10% LVEF reduction from baseline without HF symptoms, or ≥ 5% reduction with symptoms, to LVEF < 55%	EQ-5D-3L, MLHFQ (secondary)	Every 3 months during treatment; baseline through completion of trastuzumab; post-treatment VO_2_ peak assessment	Higher MVPA during cancer treatment was associated with better QoL, preserved systolic and diastolic function, and higher post-treatment cardiorespiratory fitness	GEE adjusted for age, time, anthracycline dose, cardiac radiation dose, cardiac medication, and CVD risk factors	RQ 2
Díaz-Balboa et al. Spain (2024) [38]	Randomized controlled trial (*n* = 122)	Breast cancer Adjuvant or neoadjuvant anthracyclines and/or anti-HER2 targeted therapy	Physical activity	Primary: CTRCD; LVEF, GLS. Secondary: NT-proBNP	CTRCD: LVEF decrease ≥ 10 percentage points to < 53% or GLS decrease > 15% from baseline	FACT-B, HADS, GLTEQ, PREDIMED Questionnaire (secondary)	Baseline and after completion of cardiotoxic treatment; mean 5.8 months	No CTRCD occurred; LVEF decline was attenuated while GLS and cardiac biomarkers remained stable. QoL and functional capacity remained stable	Analyses were primarily between-group comparisons using ITT/PP	RQ 3
Vincent et al. France (2020) [39]	Randomized controlled trial (*n* = 94)	Breast cancer Chemotherapy followed by radiotherapy. FEC 100 **→** paclitaxel For HER2+ patients: trastuzumab	Physical activity	VO_2_ peak assessed as a measure of cardio- respiratory fitness	Cardiotoxicity not defined	QLQ-C30 (secondary)	Baseline, 6 months, 12 months	Home-based physical activity improved cardiorespiratory fitness, functional capacity, and QoL during active exercise; VO_2_ peak declined without exercise, with no significant between-group differences	No multivariable adjusted analysis reported; repeated-measures ANOVA and between-group tests were used	RQ 4
Spector et al. USA (2014) [40]	Pilot interventional study (*n* = 13)	Breast cancer Doxorubicin, taxanes, trastuzumab	Physical activity	VO_2_ peak was assessed as a measure of cardio- respiratory fitness	Cardiotoxicity not defined	FACT-B, FACIT-F (secondary)	Baseline and post-intervention (16 weeks)	Home-based exercise improved physical activity, strength, functional movement, and cardiorespiratory fitness, with significant improvements in selected QoL domains and a nonsignificant reduction in fatigue	No adjusted analysis; Wilcoxon signed-rank tests and Spearman correlations	RQ 4
Griffith et al. USA (2009) [41]	Randomized controlled trial (*n* = 126)	Breast cancer, prostate cancer, other solid tumours Chemotherapy, radiation therapy, or a combination of them	Physical activity	VO_2_ peak was assessed as cardiorespiratory fitness	Cardiotoxicity not defined	MOS SF- 36 (secondary)	Baseline and post-intervention; 8 weeks	Walking intervention improved cardiorespiratory fitness and self-reported physical functioning, particularly in patients with prostate cancer	VO_2_ analysis adjusted for baseline VO_2_ peak and PAQ; age was examined as a predictor of physical function	RQ 4
**D. Supportive Care Studies**										
Author Country, Publication Year	Study Characteristics	Cancer Type/ Chemotherapy	Intervention/ Exposure	Cardiac Endpoint	Cardiotoxicity Definition	HRQoL Instruments, HRQoL Endpoint (Type)	Follow-Up	Main Findings	Adjusted Analysis	Study Focus
Koop et al. Netherlands (2022) [42]	Qualitative semi-structured interview (*n* = 12)	Breast cancer All: anthracycline-based, trastuzumab, surgery. Hormone receptor +: additional hormone therapy; 9 patients: radiotherapy	QoL and experiences with cardiac surveillance	CTRCD: systolic HF, MI, valvular disease, conduction disorders; LVEF at CTRCD diagnosis	Cardiologist-confirmed CTRCD; no standardized quantitative definition	QoL explored using the Positive Health framework (primary) Personal experience with cardiac surveillance (secondary)	Retrospective patient experiences; interviews conducted Jan–May 2019	CTRCD impaired physical, emotional, and social well-being, highlighting the need for personalized, multidisciplinary care to support long-term HRQoL. Effective communication between patients and healthcare professionals was also emphasized	Not applicable; qualitative thematic analysis	RQ 1

Abbreviations: PH-FECH = Paclitaxel + Trastuzumab followed by 5-Fluorouracil, Epirubicin, and Cyclophosphamide, NAC = Neoadjuvant Chemotherapy, LVEF = Left Ventricular Ejection Fraction, QoL = Quality of Life, SF-36 = Short Form (36) Health Survey, EQ-5D-5L = EuroQol 5-Dimension 5-Level questionnaire, ECOG = Eastern Cooperative Oncology Group Performance Status, ORs = Odds Ratios, LV = Left Ventricle, PWV = Pulse Wave Velocity, CMR-LGE = Cardiac Magnetic Resonance with Late Gadolinium Enhancement, BNP = B-type Natriuretic Peptide, TnI = Troponin I, MLHFQ = Minnesota Living with Heart Failure Questionnaire, CV = Cardiovascular, EC-P = Epirubicin, Cyclophosphamide → Paclitaxel, NHL = Non-Hodgkin Lymphoma, R-CHOP = Rituximab, Cyclophosphamide, Doxorubicin, Vincristine, Prednisone, HL = Hodgkin Lymphoma, GLS = Global Longitudinal Strain, EQ-5D = EuroQol 5-Dimension questionnaire, Fast-SENC CMR = Fast Strain-Encoded Cardiac Magnetic Resonance, HF = Heart Failure, HRQoL = Health-Related Quality of Life, TC = Testicular Cancer, CVD = Cardiovascular Disease, BEP = Bleomycin, Etoposide, Cisplatin, CAD = Coronary Artery Disease, MI = Myocardial Infarction, EORTC QLQ-C30 = European Organisation for Research and Treatment of Cancer Quality of Life Questionnaire—Core 30, CTCAE = Common Terminology Criteria for Adverse Events, EF = Ejection Fraction, LASA-6 = Linear Analogue Self-Assessment—6 items, OS = Overall Survival, CHF = Congestive Heart Failure, NYHA = New York Heart Association, CTRCD = Cancer Therapy–Related Cardiac Dysfunction, ACEi = Angiotensin-Converting Enzyme Inhibitors, ARB = Angiotensin II Receptor Blockers, CMR = Cardiac Magnetic Resonance, T2 = T2-weighted magnetic resonance imaging parameter, T1 = T1-weighted magnetic resonance imaging parameter, ECV = Extracellular Volume, EQ-5D-3L = EuroQol 5-Dimension 3-Level questionnaire, GEE = Generalized Estimating Equations, AC = Doxorubicin + Cyclophosphamide, LTF = Long-Term Follow-Up, MOS SF-36 = Medical Outcomes Study 36-Item Short Form Health Survey, DASI = Duke Activity Status Index, AML = Acute Myeloid Leukaemia, ALL = Acute Lymphoblastic Leukaemia, FACT = Functional Assessment of Cancer Therapy, FACT-G = Functional Assessment of Cancer Therapy—General, FACT-Lym = Functional Assessment of Cancer Therapy—Lymphoma, FACT-B = Functional Assessment of Cancer Therapy—Breast, FACIT-F = Functional Assessment of Chronic Illness Therapy—Fatigue, IPAQ = International Physical Activity Questionnaire, VO_2_ peak = Peak Oxygen Uptake, MDASI-HF = MD Anderson Symptom Inventory—Heart Failure, 7-DPARQ = 7-Day Physical Activity Recall Questionnaire, MRI = Magnetic Resonance Imaging, SD = Standard Deviation, RV = Right Ventricle, EDV = End-Diastolic Volume, ESV = End-Systolic Volume, HR = Heart Rate, DDAC = Dose-Dense Doxorubicin and Cyclophosphamide, QLQ-FA12 = EORTC Quality of Life Questionnaire—Fatigue Module (12 items), E/A = Early-to-late ventricular filling velocity ratio, E/e′ = Ratio of early mitral inflow velocity to early diastolic mitral annular velocity, MVPA = Moderate-to-Vigorous Physical Activity, NT-proBNP = N-terminal pro–B-type Natriuretic Peptide, HADS = Hospital Anxiety and Depression Scale, GLTEQ = Godin Leisure-Time Exercise Questionnaire, PREDIMED Questionnaire = PREvención con DIeta MEDiterránea, ITT = Intention-to-Treat, PP = Per-Protocol, FEC-100 = Fluorouracil + Epirubicin + Cyclophosphamide (100 mg/m^2^ epirubicin dose), PAQ = Physical Activity Questionnaire.

**Table 2:** Physical activity regimens in the prevention of chemotherapy-induced cardiotoxicity.

No.	Author, Country, Publication Year	Cancer Type (Sample)	Age	Intervention	Details
1.	Heinze-Milne et al. Canada (2021) [32]	Breast cancer, haematological cancer (*n* = 10)	49.8 ± 13.0	Supervised aerobic training (35–85% HRR), 20–45 min/session, 2×/week × 12 weeks	HRR zones used during treadmill/cycle training supervised by staff. 20–45 min per session with warm-up/cool-down included
2.	Hughes et al. USA (2011) [33]	Hodgkin Lyphoma, Leukaemia (*n* = 2)	Pt 1, 56 Pt 2, 46	Individualized aerobic training (progressive RPE ≥ 12 to target 50% HRR), 20–30 min/session, 3×/week × 16 weeks (supervised + home-based).	RPE Started with shorter bouts (~11 min) and progressed duration and intensity over time. The program aimed for ≥30 min continuous exercise per session at ~50% HRR or RPE ≥ 12.
3.	Costa et al. USA (2025) [34]	NHL, HL (*n* = 20)	PAI, 50.7 (18.4) HLI, 57.3 (13.6)	PAI—Tailored supervised + home-based multicomponent training (15 min warm-up + 20 min resistance + 15 min aerobic + 10 min cool-down), 2×/week × 6 months (centre- or home-based). HLI—control group receiving bi-weekly healthy lifestyle education (e.g., nutrition, stress management) without direct physical activity prescription. Randomization ratio: 2:1 (PAI:HLI)	The intervention was individualized and progressive, combining aerobic and resistance exercise Sessions were supervised by exercise physiologists but adapted to home-based delivery when needed (ex. during COVID-19). Behavioural support components as part of the design are also included; (PAI) was grounded in behavioural theory.
4.	Murray et al. Australia (2023) [35]	Breast cancer (*n* = 5)	Pt 1, 68, Pt 2, 38, Pt 3, 55, Pt 4, 45, Pt 5, 52	Choice between supervised, individualized aerobic exercise intervention *or* receiving standard care without structured exercise. Individualized supervised aerobic training (30 min continuous cycling at 60–70% HR_peak_, 3×/week × 8 weeks).	Moderate intensity aerobic exercise prescribed based on each participant’s peak heart rate from baseline exercise testing. Intensity was adjusted around treatment cycles (lower post-chemotherapy, higher mid-cycle).
5.	Schneider et al. Switzerland (2024) [36]	Breast cancer, lymphoma, blood cancer, other (*n* = 262)	52 (40–61)	Aerobic + strength, individualized, supervised during or after chemotherapy, frequency not specified × variable duration	Compared exercise training concurrent with chemotherapy versus exercise after chemotherapy completion, rather than fixed session prescriptions.
6.	Peck et al., Canada (2022) [37]	Breast cancer (*n* = 88)	51.4 ± 8.9	Self-reported MVPA ≥ 90 min/week during sequential anthracycline + Trastuzumab	Does not involve a structured exercise intervention prescribed by the investigators. It assessed self-reported levels of physical activity (MVPA)
7.	Díaz-Balboa et al. Spain (2024) [38]	Breast cancer (*n* = 122)	48.87 (8.24)	Supervised exercise-based cardio-oncology rehabilitation (aerobic + resistance, 50–85% HRR), 60 min/session, 2×/week × ~5.8 months (during anthracycline ±HER2 therapy).	~ 60 min = Warm-up, Aerobic Training, Resistance/Strength Training, Cool-down/Flexibility
8.	Vincent et al. France (2020) [39]	Breast cancer (*n* = 94)	53.0 ± 8.9	Home-based adapted PA (1 resistance + 2 aerobic sessions/week; aerobic on bicycle ergometer 3×8 min @ 60% max aerobic power progressing to 30 min @ 70%, plus weekly resistance with elastic bands), 3×/week × 6–12 months (timing: during vs. after chemo vs. during + after).	Combined aerobic + resistance home-based exercise tailored from baseline cardiopulmonary testing. The program was randomized into 3 arms based on timing relative to chemotherapy (during, after, or both).
9.	Spector et al. USA (2014) [40]	Breast cancer (*n* = 13)	51.6	Home-based progressive aerobic (@ 40–65% HRR) + resistance training, motivational support, 16 weeks.	Motivational component: weekly counselling calls and exercise logs to support adherence.
10.	Griffith et al. USA (2009) [41]	Breast cancer, prostate cancer, other solid tumours (*n* = 126)	60.2 (10.6)	Home-based brisk walking (20–30 min @ ~50–70% HRmax + 5 min cool-down), 5×/week × duration of cancer treatment (~8–16 weeks) (randomized vs. usual care).	Participants received biweekly telephone support from study staff to check progress and encouragement, but no supervised facility sessions.

Abbreviations: HRR = Heart Rate Reserve, Pt = Patient, RPE = Rate of Perceived Exertion, PAI = Physical Activity Intervention, HLI = Healthy Living Intervention, HR_peak_ = Peak Heart Rate, MVPA = Moderate-to-Vigorous Physical Activity, HRR = Heart Rate Reserve, HER2 = Human Epidermal Growth Factor Receptor 2, PA = Physical Activity, HRmax = Maximum Heart Rate.

## Data Availability

No new data were created or analyzed in this study. Data sharing is not applicable to this article.

## Supplementary Materials

The following supporting information can be downloaded at: https://www.mdpi.com/article/10.3390/jcm15176760/s1, Section S1: Search query; Section S2: Classification of Instruments Used in HRQOL and Related Domains; Table S1: General HRQOL Instruments; Table S2: Disease-Specific HRQOL Instruments; Table S3: Concomitant/HRQOL-Related (Indirect or Proxy Instruments).

## References

[1] Siegel R.L., Giaquinto A.N., Jemal A. (2024). Cancer statistics, 2024. CA Cancer J. Clin..

[2] Sung H., Ferlay J., Siegel R.L., Laversanne M., Soerjomataram I., Jemal A., Bray F. (2021). Global cancer statistics 2020: GLOBOCAN estimates of incidence and mortality worldwide for 36 cancers in 185 countries. CA Cancer J. Clin..

[3] Topalian S.L., Drake C.G., Pardoll D.M. (2015). Immune checkpoint blockade: A common denominator approach to cancer therapy. Cancer Cell.

[4] June C.H., O’Connor R.S., Kawalekar O.U., Ghassemi S., Milone M.C. (2018). CAR T cell immunotherapy for human cancer. Science.

[5] Haslam A., Prasad V. (2019). Estimation of the percentage of US patients with cancer who are eligible for and respond to checkpoint inhibitor immunotherapy drugs. JAMA Netw. Open.

[6] IQVIA Institute for Human Data Science (2023). Global Oncology Trends 2023.

[7] Henry D.H., Viswanathan H.N., Elkin E.P., Traina S., Wade S., Cella D. (2008). Symptoms and treatment burden associated with cancer treatment: Results from a cross-sectional national survey in the U.S. Support. Care Cancer.

[8] Cardinale D., Colombo A., Bacchiani G., Tedeschi I., Meroni C.A., Veglia F., Civelli M., Lamantia G., Colombo N., Curigliano G. (2015). Early detection of anthracycline cardiotoxicity and improvement with heart failure therapy. Circulation.

[9] Ky B. (2023). Growing, Building, and Defining the Field of Cardio-Oncology for Our Patients. JACC CardioOncol..

[10] Ky B. (2024). Priorities in Cardio-Oncology: Filling the Gaps in Evidence. JACC CardioOncol..

[11] Lyon A.R., López-Fernández T., Couch L.S., Asteggiano R., Aznar M.C., Bergler-Klein J., Boriani G., Cardinale D., Cordoba R., Cosyns B. (2022). 2022 ESC Guidelines on cardio-oncology developed in collaboration with the European Hematology Association (EHA), the European Society for Therapeutic Radiology and Oncology (ESTRO) and the International Cardio-Oncology Society (ICOS). Eur. Heart J..

[12] Fradley M.G., Wilcox N., Frain I., Rao V.U., Carver J., Guha A., Dent S. (2023). Developing a Clinical Cardio-Oncology Program and the Building Blocks for Success: JACC: CardioOncology How To. JACC CardioOncol..

[13] Jacobs L.A., Shulman L.N. (2024). Cardio-Oncology Care Delivery for All Patients With Cancer Within Academic and Community Settings. JACC CardioOncol..

[14] Cardinale D., Sandri M.T., Colombo A., Colombo N., Boeri M., Lamantia G., Civelli M., Peccatori F., Martinelli G., Fiorentini C. (2004). Prognostic value of troponin I in cardiac risk stratification of cancer patients undergoing high-dose chemotherapy. Circulation.

[15] Thavendiranathan P., Poulin F., Lim K.D., Plana J.C., Woo A., Marwick T.H. (2014). Use of myocardial strain imaging by echocardiography for the early detection of cardiotoxicity in patients during and after cancer chemotherapy: A systematic review. J. Am. Coll. Cardiol..

[16] Kalam K., Marwick T.H. (2013). Role of cardioprotective therapy for prevention of cardiotoxicity with chemotherapy: A systematic review and meta-analysis. Eur. J. Cancer.

[17] Swain S.M., Whaley F.S., Ewer M.S. (2003). Congestive heart failure in patients treated with doxorubicin: A retrospective analysis of three trials. Cancer.

[18] Cella D., Choi S., Garcia S., Cook K.F., Rosenbloom S., Lai J.-S., Tatum D.S., Gershon R. (2014). Setting standards for severity of common symptoms in oncology using the PROMIS item banks and expert judgment. Qual. Life Res..

[19] Hwang I.C., Park S.M., Yun Y.H., Kim K.K. (2015). Gaps in Health-Related Quality of Life Among Survivors of Cancer and Cardiovascular Disease: The Korean National Health and Nutrition Examination Survey, 2005 (KNHANES III). Asia Pac. J. Public Health.

[20] Buziashvili Y.I., Stilidi I.S., Asymbekova E.U., Matskeplishvili S.T., Tugeeva E.F., Akhmedyarova N.K., Artamonova E.V., Akildzhonov F.R. (2024). Comprehensive assessment of the quality of life in patients with breast cancer during neoadjuvant chemotherapy. Heart Vessel. Transplant..

[21] Drafts B.C., Twomley K.M., D’Agostino R., Lawrence J., Avis N., Ellis L.R., Thohan V., Jordan J., Melin S.A., Torti F.M. (2013). Low to moderate dose anthracycline-based chemotherapy is associated with early noninvasive imaging evidence of subclinical cardiovascular disease. JACC Cardiovasc. Imaging.

[22] Geršak B.M., Kukec A., Steen H., Montenbruck M., Šoštarič M., Schwarz A.K., Esch S., Kelle S., Giusca S., Korosoglou G. (2021). Relationship between quality of life indicators and cardiac status indicators in chemotherapy patients. Slov. J. Public Health.

[23] Lubberts S., Groot H.J., de Wit R., Mulder S., Witjes J.A., Kerst J.M., Groenewegen G., Lefrandt J.D., van Leeuwen F.E., Nuver J. (2023). Cardiovascular disease in testicular cancer survivors: Identification of risk factors and impact on quality of life. J. Clin. Oncol..

[24] Huang G., Hua S., Liu H., Zhou H., Chen X., Wang Z., Yu W. (2022). Efficacy of ifosfamide combined with liposome doxorubicin on osteosarcoma and its effects on serum IL-10, TNF-α, and IFN-γ in patients with osteosarcoma. Am. J. Transl. Res..

[25] Sideras K., Hillman D.W., Giridhar K., Ginos B.F., Tenglin R.C., Liu H., Chen B., Tan W., Gross G.G., Mowat R.B. (2022). HeRandomized phase II study of two doses of pixantrone in patients with metastatic breast cancer (NCCTG N1031, Alliance). Oncologist.

[26] Conte P.F., Guarneri V., Bruzzi P., Prochilo T., Salvadori B., Bolognesi A., Aldrighetti D., Venturini M., Rosso R., Mammoliti S. (2004). Concomitant versus sequential administration of epirubicin and paclitaxel. Cancer.

[27] Suntheralingam S., Osataphan N., Power C., Fan C.-P.S., Abdel-Qadir H., Amir E., Thavendiranathan P. (2024). Safety of continuing trastuzumab for mild cardiotoxicity: A cardiovascular magnetic resonance imaging study. CJC Open.

[28] Ganz P.A., Romond E.H., Cecchini R.S., Rastogi P., Geyer C.E., Swain S.M., Jeong J.-H., Fehrenbacher L., Gross H.M., Brufsky A.M. (2017). Long-term follow-up of cardiac function and quality of life for patients in NSABP Protocol B-31/NRG Oncology. J. Clin. Oncol..

[29] Sangha O., Stucki G., Liang M.H., Fossel A.H., Katz J.N. (2003). The self-administered comorbidity questionnaire: A new method to assess comorbidity for clinical and health services research. Arthritis Rheum..

[30] Ganz P.A., Day R., Ware J.E., Redmond C., Fisher B. (1995). Base-line quality-of-life assessment in the National Surgical Adjuvant Breast and Bowel Project Breast Cancer Prevention Trial. J. Natl. Cancer Inst..

[31] Skrypnyk I., Maslova G., Lymanets T., Gusachenko I. (2019). How to Improve Quality of Life in Patients with Acute Leukemia and Comorbid Ischemic Heart Disease Treated with Anthracycline-Based Induction Chemotherapy. Exp. Oncol..

[32] Heinze-Milne S.D., Keats M.R., Blanchard C., Giacomantonio N., MacDonald D., Rajda M., Younis T., Grandy S.A. (2021). Exercise to Prevent Anthracycline-Based Cardiotoxicity (EXACT): A Feasibility Study. Transl. J. Am. Coll. Sports Med..

[33] Hughes D.C., Lenihan D.J., Harrison C.A., Basen-Engquist K.M. (2011). Exercise intervention for cancer survivors with heart failure: Two case reports. J. Exerc. Sci. Fit..

[34] Costa J.V., Lucas A.R., Mihalko S.L., Brubaker P.H., Marshall A., Leitzelar B., Wolle B.R., Norton S., Franco R.L., Via J. (2025). Physical activity intervention in lymphoma patients. Pilot Feasibility Stud..

[35] Murray J., Bennett H., Selva-Nayagam S., Joshi R., Bezak E., Perry R. (2023). Impact of aerobic training on cardiovascular function, fitness, and patient reported outcomes during anthracycline chemotherapy. Integr. Cancer Ther..

[36] Schneider C., Dierks A., Rabaglio M., Campbell K.L., Wilhelm M., Eser P. (2024). Timing of cardio-oncological rehabilitation and cardiorespiratory fitness in patients receiving cardiotoxic chemotherapy. Swiss Med. Wkly..

[37] Peck S.S., Esmaeilzadeh M., Rankin K., Shalmon T., Fan C.-P.S., Somerset E., Amir E., Thampinathan B., Walker M., Sabiston C.M. (2022). Self-reported physical activity, quality of life, cardiac function, and cardiorespiratory fitness in women with HER2+ breast cancer. JACC CardioOncol..

[38] Díaz-Balboa E., Peña-Gil C., Rodríguez-Romero B., Cuesta-Vargas A.I., Lado-Baleato O., Martínez-Monzonís A., Pedreira-Pérez M., Palacios-Ozores P., López-López R., González-Juanatey J.R. (2024). Exercise-based cardio-oncology rehabilitation for cardiotoxicity prevention. Prog. Cardiovasc. Dis..

[39] Vincent F., Deluche E., Bonis J., Leobon S., Antonini M.-T., Laval C., Favard F., Dobbels E., Lavau-Denes S., Labrunie A. (2020). Home-Based Physical Activity in Patients With Breast Cancer: During and/or After Chemotherapy? Impact on Cardiorespiratory Fitness. A 3-Arm Randomized Controlled Trial (APAC). Integr. Cancer Ther..

[40] Spector D., Deal A.M., Amos K.D., Yang H., Battaglini C.L. (2014). Home-based motivational exercise program for African American breast cancer survivors. Integr. Cancer Ther..

[41] Griffith K., Wenzel J., Shang J.J., Thompson C., Stewart K., Mock V. (2009). Impact of a Walking Intervention on Cardiorespiratory Fitness, Self-Reported Physical Function, and Pain in Patients Undergoing Treatment for Solid Tumors. Cancer.

[42] Koop Y., van Zadelhof N., Maas A.H.E.M., Atsma F., El Messaoudi S., Vermeulen H. (2022). Quality of life in breast cancer patients with cardiac dysfunction. Eur. J. Cardiovasc. Nurs..

[43] Lin K.J., Lengacher C.A. (2019). Anthracycline chemotherapy-induced cardiotoxicity in breast cancer survivors: A systematic review. Oncol. Nurs. Forum.

[44] Hershman D.L., Shao T. (2009). Anthracycline cardiotoxicity after breast cancer treatment. Oncology.

[45] Ware J.E., Sherbourne C.D. (1992). The MOS 36-item short-form health survey (SF-36): I. Conceptual framework and item selection. Med. Care.

[46] Vasbinder A., Wadden E., Cheng R.K., Barac A., Friese C.R., Sun Y., Shadyab A.H., Liu L., Martin L.W., Stefanick M. (2025). Relationship between incident cardiovascular disease and quality of life after a breast cancer diagnosis. J. Cancer Surviv..

[47] Bloom M.W., Vo J.B., Rodgers J.E., Ferrari A.M., Nohria A., Deswal A., Cheng R.K., Kittleson M.M., Upshaw J.N., Palaskas N. (2025). Cardio-Oncology and Heart Failure: A Scientific Statement From the Heart Failure Society of America. J. Card. Fail..

[48] Keramida K., Lopez-Fernandez T., Anker M.S., Petrie M.C., Ameri P., Anderson L.J., Bergler-Klein J., Deswal A., Farmakis D., Ibañez B. (2025). Heart failure with preserved ejection fraction in cancer patients and survivors. A scientific statement of the Heart Failure Association of the ESC and the ESC Council of Cardio-Oncology. Eur. J. Heart Fail..

[49] Rakisheva A., Farmakis D., Attanasio A., Bayes Genis A., Cohen-Solal A., Gulati G., Halle M., Hill L., Lopez Fernandez T., Lyon A.R. (2025). Prevention of cancer therapy-related cardiac dysfunction and heart failure in cancer patients and survivors. A Clinical Consensus Statement of the Heart Failure Association, the European Association of Preventive Cardiology of the ESC, and the ESC Council of Cardio-Oncology. Eur. J. Heart Fail..

[50] Sartorio A., Cristin L., Dal Pont C., Farzaneh-Far A., Romano S. (2025). Global longitudinal strain as an early marker of cardiac damage after cardiotoxic medications, a state-of-the-art review. Prog. Cardiovasc. Dis..

[51] Qiu S., Zhang Y., Hou Y., Chen S., Yu H., Li H., Zhao L., Zhang X., Zhang X., Liu J. (2025). Integrative speckle-tracking echocardiography for the early detection and prediction of cancer therapy-related cardiac dysfunction. Cardio-Oncology.

[52] Camilli M., Ballacci F., Lamendola P., Viscovo M., Tamburrini G., Tinti L., Torre I., Amore L., Hohaus S., Crea F. (2025). Strain-derived myocardial work indices in adult cancer survivors: Results from an observational study and comparison with available reference ranges. Cardio-Oncology.

[53] Migliari M., Fazzini L., Campana N., Deidda M., Dessì M., Cadeddu Dessalvi C. (2025). Current strategies for prevention of cancer therapy-related cardiotoxicity: Pharmacological, non-pharmacological and emerging approaches. Front. Cardiovasc. Med..

[54] Zhang D., Xiong X., Ding H., He X., Li H., Yao Y., Ma R., Liu T. (2025). Effectiveness of Exercise-Based Interventions in Preventing Cancer Therapy-Related Cardiac Dysfunction in Patients with Breast Cancer: A Systematic Review and Network Meta-Analysis. Int. J. Nurs. Stud..

[55] Marmol-Perez A., Gracia-Marco L., Clavero-Jimeno A., Amaro-Gahete F.J., Ruiz J.R., Carneiro-Barrera A. (2025). Effects of Exercise-Based Interventions on Health-Related Quality of Life in Adults after Cancer: A Systematic Review and Meta-Analysis. Ann. Phys. Rehabil. Med..

[56] Adams S.C., Rivera-Theurel F., Scott J.M., Nadler M.B., Foulkes S., Leong D., Nilsen T., Porter C., Haykowsky M., Abdel-Qadir H. (2025). Cardio-oncology rehabilitation and exercise: Evidence, priorities, and research standards from the ICOS-CORE working group. Eur. Heart J..

[57] Scott J.M., Zabor E.C., Schwitzer E., Koelwyn G.J., Adams S.C., Nilsen T.S., Moskowitz C.S., Matsoukas K., Iyengar N.M., Dang C.T. (2018). Efficacy of Exercise Therapy on Cardiorespiratory Fitness in Patients With Cancer: A Systematic Review and Meta-Analysis. J. Clin. Oncol..

[58] Cormie P., Zopf E.M., Zhang X., Schmitz K.H. (2017). The Impact of Exercise on Cancer Mortality, Recurrence, and Treatment-Related Adverse Effects. Epidemiol. Rev..

[59] Schmitz K.H., Campbell A.M., Stuiver M.M., Pinto B.M., Schwartz A.L., Morris G.S., Ligibel J.A., Cheville A., Galvão D.A., Alfano C.M. (2019). Exercise Is Medicine in Oncology: Engaging Clinicians to Help Patients Move through Cancer. CA Cancer J. Clin..

[60] Sánchez B., González P., Goveo I., Contreras P., Dominguez L., Manrique P., Vargas M. (2025). Chemotherapy-Induced Cardiotoxicity: Mechanisms, Detection and Emerging Therapies in Cardio-Oncology. Discoveries.

[61] Gheorghe G.S., Ciobanu A., Hodorogea A.S., Gheorghe A.C.D., Scăunașu R.V., Nanea I.T., Stoian M.I., Ilieşiu A.M. (2021). Cardiotoxicity of Anticancer Therapies: Focus on the Role of the Cardio-Oncological Team. A Practical Review. Farmacia.

[62] Niklasson A., Maher J., Patil R., Sillén H., Chen J., Gwaltney C., Rydén A. (2022). Living with heart failure: Patient experiences and implications for physical activity and daily living. ESC Heart Fail..

[63] Neilan T.G., Quinaglia T., Onoue T., Mahmood S.S., Drobni Z.D., Gilman H.K., Smith A., Heemelaar J.C., Brahmbhatt P., Ho J.S. (2023). Atorvastatin for Anthracycline-Associated Cardiac Dysfunction: The STOP-CA Randomized Clinical Trial. JAMA.

[64] Omland T., Heck S.L., Gulati G. (2022). The Role of Cardioprotection in Cancer Therapy Cardiotoxicity: JACC: CardioOncology State-of-the-Art Review. JACC CardioOncol..

[65] Simela C., Walker J.M., Ghosh A.K., Chen D.H. (2025). SGLT2 inhibitors for prevention and management of cancer treatment-related cardiovascular toxicity: A review of potential mechanisms and clinical insights. Cardio-Oncology.

[66] Mir A., Badi Y., Bugazia S., Nourelden A.Z., Fathallah A.H., Ragab K.M., Alsillak M., Elsayed S.M., Hagrass A.I., Bawek S. (2023). Efficacy and Safety of Cardioprotective Drugs in Chemotherapy-Induced Cardiotoxicity: An Updated Systematic Review & Network Meta-Analysis. Cardio-Oncology.

[67] Lyon A.R., Dent S., Stanway S., Earl H., Brezden-Masley C., Cohen-Solal A., Tocchetti C.G., Moslehi J.J., Groarke J.D., Bergler-Klein J. (2020). Baseline Cardiovascular Risk Assessment in Cancer Patients Scheduled to Receive Cardiotoxic Cancer Therapies: A Position Statement and New Risk Assessment Tools from the Cardio-Oncology Study Group of the Heart Failure Association of the European Society of Cardiology in Collaboration with the International Cardio-Oncology Society. Eur. J. Heart Fail..

[68] Alexandre J., Cautela J., Ederhy S., Damaj G.L., Salem J.-E., Barlesi F., Farnault L., Charbonnier A., Mirabel M., Champiat S. (2020). Cardiovascular Toxicity Related to Cancer Treatment: A Pragmatic Approach to the American and European Cardio-Oncology Guidelines. J. Am. Heart Assoc..

[69] Travers S., Alexandre J., Baldassarre L.A., Salem J.E., Mirabel M. (2025). Diagnosis of Cancer Therapy-Related Cardiovascular Toxicities: A Multimodality Integrative Approach and Future Developments. Arch. Cardiovasc. Dis..

[70] López-Fernández T., van der Meer P., Lyon A.R. (2025). An Update on the European Society of Cardiology Council of Cardio-Oncology. Eur. Heart J..

[71] Rao V.U., Deswal A., Lenihan D., Dent S., Lopez-Fernandez T., Lyon A.R., Barac A., Palaskas N., Chen M.H., Villarraga H.R. (2025). Quality-of-Care Measures for Cardio-Oncology: An IC-OS and ACC Cardio-Oncology Leadership Council Perspective. JACC CardioOncol..

[72] Leong D.P., Cosman T., Alhussein M.M., Tyagi N.K., Karampatos S., Barron C.C., Wright D., Tandon V., Magloire P., Joseph P. (2019). Safety of Continuing Trastuzumab Despite Mild Cardiotoxicity: A Phase I Trial. JACC CardioOncol..

[73] Ignatenko G.A., Sedakov I.E., Kolycheva O.V., Kaganov O.I., Orlov A.E., Bondarenko N.N. (2024). Treatment of Cardiotoxicity in Patients with Invasive Breast Cancer during Neoadjuvant Chemotherapy. Sci. Innov. Med..

[74] Chiang C.H., Chang Y.C., Haw Y., Tan J.Y., Chiang C.-H., Hsia Y.P., Chiang C.-H. (2024). Exercise and cardiotoxicity meta-analysis. Oncology.

[75] Wang J., Liu S., Meng X., Zhao X., Wang T., Lei Z., Lehmann H.I., Li G., Alcaide P., Bei Y. (2024). Exercise Inhibits Doxorubicin-Induced Cardiotoxicity via Regulating B Cells. Circ. Res..

[76] Bognár S.A., Teutsch B., Bunduc S., Veres D.S., Szabó B., Fogarasi B., Zahariev O.J., Vörhendi N., Almog O., Hadani Y. (2024). Psychological intervention improves quality of life in patients with early-stage cancer: A systematic review and meta-analysis of randomized clinical trials. Sci. Rep..

[77] Dils A.T., O’Keefe K., Dakka N., Azar M., Chen M., Zhang A. (2024). The efficacy of cognitive behavioral therapy for mental health and quality of life among individuals diagnosed with cancer: A systematic review and meta-analysis. Cancer Med..

[78] Wang X., Chen L., Xu Z., Zhu C., Tang Y., Sun J., Zhu L. (2025). Effects of a Multi-Component Positive Psychological Intervention on Negative Emotions, Fatigue and Quality of Life in Patients with Breast Cancer during Initial Chemotherapy: A Randomized Controlled Trial. Eur. J. Oncol. Nurs..

[79] Böger R.H. (2007). The pharmacodynamics of L-arginine. J. Nutr..

[80] Tousoulis D., Böger R.H., Antoniades C., Siasos G., Stefanadi E., Stefanadis C. (2007). Mechanisms of Disease: L-Arginine in Coronary Atherosclerosis—A Clinical Perspective. Nat. Rev. Cardiol..

[81] Hull S.C., Mszar R., Ostfeld R.J., Ferrucci L.M., Mucci L.A., Giovannucci E., Loeb S. (2025). Diet and Prevention of Cardiovascular Disease and Cancer: JACC: CardioOncology State-of-the-Art Review. JACC CardioOncol..

[82] Boriani G., Imberti J.F., Asteggiano R., Ameri P., Mei D.A., Farkowski M., Chun J., Merino J.L., López-Fernández T., Lyon A.R. (2025). Mobile/wearable digital devices for care of active cancer patients: A survey from the ESC Council of Cardio-Oncology. Eur. Heart J. Digit. Health.

