# Supplementary material for: Balancing Oncological Care and Quality of Life: Diagnostic and Therapeutic Strategies for Chemotherapy-Induced Cardiotoxicity—A Narrative Review
Source: J Clin Med. 2026 Aug 31;15(17):6760. doi: 10.3390/jcm15176760 (PMC13566244; doi:10.3390/jcm15176760)
Supplement: Supplementary file 1 [file jcm-15-06760-s001.zip › jcm-4478049-supplementary.pdf]

Section S1

Search query

PubMed: (("quality of life" or "QOL" or "HRQOL" or "health-related quality of life") and ("cardiotoxicity" or "CTRCD" or "cancer therapy-related cardiac dysfunction") and ("cancer" or "neoplasm"));

Web of Science: (("quality of life" or "QOL" or "HRQOL" or "health-related quality of life") and ("cardiotoxicity" or "CTRCD" or "cancer treatment-related cardiac dysfunction") and ("cancer" or "neoplasm"));

Scopus: TITLE-ABS ("quality of life" OR "QOL" OR "HRQOL" OR "health-related quality of life") AND ("cardiotoxicity" OR "CTRCD" OR "cancer therapy-related cardiac dysfunction") AND ("cancer" OR "neoplasm"))

Section S2

Classification of Instruments Used in HRQOL and Related Domains

Table S1. General HRQOL Instruments

Instrument	Subcategory	Notes
EQ-5D / EQ-5D-3L / EQ-5D-5L	Generic utility-based	Standard preference-based instrument applicable across diseases
SF-36 / MOS SF-36	Generic health status	Measures physical and mental health domains in general populations

Abbreviations: EQ-5D = EuroQol 5-Dimension questionnaire, EQ-5D-3L = EuroQol 5-Dimension 3-Level questionnaire, EQ-5D-5L = EuroQol 5-Dimension 5-Level questionnaire, SF-36 = Short Form (36) Health Survey, MOS SF-36 = Medical Outcomes Study 36-Item Short Form Health Survey

Table S2. Disease-Specific HRQOL Instruments

Instrument	Subcategory	Notes
MLHFQ	Cardiology	HRQoL tool specific for heart failure
MDASI-HF	Cardiology	Symptom burden + functional impact in heart failure
QLQ-C30	Oncology (core)	Core EORTC HRQoL instrument for cancer patients
QLQ-FA12	Oncology (fatigue)	Fatigue-specific module of EORTC system
FACT-G	Oncology (general cancer)	Core cancer HRQoL questionnaire
FACT-Lym	Onco-hematology	Lymphoma-specific module
FACT-B	Oncology (breast cancer)	Breast cancer-specific HRQoL module
FACIT-F	Oncology (fatigue)	Fatigue-focused instrument widely used in cancer

Abbreviations: MLHFQ = Minnesota Living with Heart Failure Questionnaire, HRQoL = Health-related Quality of Life, MDASI-HF = MD Anderson Symptom Inventory – Heart Failure, QLQ-C30 = Quality of Life Questionnaire – Core 30, EORTC = European Organisation for Research and Treatment of Cancer, QLQ FA-12 = Quality of Life Questionnaire – Fatigue Module (12 items), FACT – G = Functional Assessment of Cancer Therapy – General, FACT-Lym = Functional Assessment of Cancer Therapy – Lymphoma, FACT – B = Functional Assessment of Cancer Therapy – Breast, FACIT-F = Functional Assessment of Chronic Illness Therapy – Fatigue

Table S3. Concomitant / HRQOL-Related (Indirect or Proxy Instruments)

Instrument	Type	Subcategory	Notes
Karnofsky Performance Status	Functional	Performance status	Global functional capacity, not direct HRQOL
ECOG Performance Status	Functional	Oncology performance	Functional staging in cancer care
DASI	Functional	Cardiopulmonary capacity	Estimates functional exercise capacity (VO_2-related)

LASA-6	Symptom / QoL proxy	Visual analogue scale	Brief self-reported QoL and symptom assessment
Cardiac symptoms checklist	Symptom-specific	Cardiology	Focused on cardiac symptom burden
Comorbidity questionnaire	Clinical burden	Comorbidities	Indirect determinant of HRQoL
HADS	Psychological	Anxiety/depression	Mental health component of HRQoL
GLTEQ	Physical activity	Leisure-time activity	Estimates activity level
IPAQ, PAQ, 7-DPARQ	Safety screening	Exercise readiness	Pre-exercise screening tool
PREDIMED	Lifestyle	Diet quality	Assesses mediterranean diet adherence

Abbreviations: HRQoL = Health-related Quality of Life, ECOG = Eastern Cooperative Oncology Group, DASI = Duke Activity Status Index, LASA – 6 = Linear Analogue Self-Assessment – 6 items, QoL = Quality of life, MDASI = MD Anderson Symptom Inventory, HADS = Hospital Anxiety and Depression Scale, GLTEQ = Godin Leisure-Time Exercise Questionnaire, IPAQ = International Physical Activity Questionnaire, PAQ = Physical Activity Questionnaire, 7-DPARQ = 7-Day Physical Activity Recall Questionnaire, PREDIMED = PREvención con DIeta MEDiterránea
